# The *Caenorhabditis elegans* HEN1 Ortholog, HENN-1, Methylates and Stabilizes Select Subclasses of Germline Small RNAs

**DOI:** 10.1371/journal.pgen.1002617

**Published:** 2012-04-19

**Authors:** Allison C. Billi, Amelia F. Alessi, Vishal Khivansara, Ting Han, Mallory Freeberg, Shohei Mitani, John K. Kim

**Affiliations:** 1 Life Sciences Institute, University of Michigan, Ann Arbor, Michigan, United States of America; 2 Department of Human Genetics, University of Michigan, Ann Arbor, Michigan, United States of America; 3 Department of Cell and Developmental Biology, University of Michigan, Ann Arbor, Michigan, United States of America; 4 Department of Bioinformatics, University of Michigan, Ann Arbor, Michigan, United States of America; 5 Core Research for Evolutional Science and Technology (CREST), Japan Science and Technology Agency (JST), Tokyo, Japan; 6 Department of Physiology, Tokyo Women's Medical University School of Medicine, Tokyo, Japan; Stanford University Medical Center, United States of America

## Abstract

Small RNAs regulate diverse biological processes by directing effector proteins called Argonautes to silence complementary mRNAs. Maturation of some classes of small RNAs involves terminal 2′-O-methylation to prevent degradation. This modification is catalyzed by members of the conserved HEN1 RNA methyltransferase family. In animals, Piwi-interacting RNAs (piRNAs) and some endogenous and exogenous small interfering RNAs (siRNAs) are methylated, whereas microRNAs are not. However, the mechanisms that determine animal HEN1 substrate specificity have yet to be fully resolved. In *Caenorhabditis elegans*, a HEN1 ortholog has not been studied, but there is evidence for methylation of piRNAs and some endogenous siRNAs. Here, we report that the worm HEN1 ortholog, HENN-1 (*HEN* of *N*ematode), is required for methylation of *C. elegans* small RNAs. Our results indicate that piRNAs are universally methylated by HENN-1. In contrast, 26G RNAs, a class of primary endogenous siRNAs, are methylated in female germline and embryo, but not in male germline. Intriguingly, the methylation pattern of 26G RNAs correlates with the expression of distinct male and female germline Argonautes. Moreover, loss of the female germline Argonaute results in loss of 26G RNA methylation altogether. These findings support a model wherein methylation status of a metazoan small RNA is dictated by the Argonaute to which it binds. Loss of *henn-1* results in phenotypes that reflect destabilization of substrate small RNAs: dysregulation of target mRNAs, impaired fertility, and enhanced somatic RNAi. Additionally, the *henn-1* mutant shows a weakened response to RNAi knockdown of germline genes, suggesting that HENN-1 may also function in canonical RNAi. Together, our results indicate a broad role for HENN-1 in both endogenous and exogenous gene silencing pathways and provide further insight into the mechanisms of HEN1 substrate discrimination and the diversity within the Argonaute family.

## Author Summary

Small RNAs serve as sentinels of the genome, policing activity of selfish genetic elements, modulating chromatin dynamics, and fine-tuning gene expression. Nowhere is this more important than in the germline, where endogenous small interfering RNAs (endo-siRNAs) and Piwi-interacting RNAs (piRNAs) promote formation of functional gametes and ensure viable, fertile progeny. Small RNAs act primarily by associating with effector proteins called Argonautes to direct repression of complementary mRNAs. HEN1 methyltransferases, which methylate small RNAs, play a critical role in accumulation of these silencing signals. In this study, we report that the 26G RNAs, a class of *C. elegans* endo-siRNAs, are differentially methylated in male and female germlines. 26G RNAs derived from the two germlines are virtually indistinguishable, except that they associate with evolutionarily divergent Argonautes. Our data support a model wherein the methylation status and, consequently, stability of a small RNA are determined by the associated Argonaute. Therefore, selective expression of Argonautes that permit or prohibit methylation may represent a new mechanism for regulating small RNA turnover. As we observe this phenomenon in the germline, it may be particularly pertinent for directing inheritance of small RNAs, which can carry information not encoded in progeny DNA that is essential for continued transgenerational genome surveillance.

## Introduction

Argonautes are an evolutionarily conserved family of proteins implicated in diverse cellular processes. They function as effector proteins in the RNA-induced silencing complex (RISC), a gene regulatory complex that binds small, non-coding RNAs to target its silencing effects. Small RNAs are broadly segregated into groups that differ in their mechanisms of biogenesis and silencing, as well as in the subsets of Argonaute effectors that bind them. The microRNAs (miRNAs) are highly conserved small RNAs processed from endogenous hairpin precursors that regulate networks of mRNAs primarily through post-transcriptional repression [1], [2]. The piRNAs, so named for the Piwi Argonautes that bind them, function predominantly in maintenance of germline integrity, often through repression of repetitive transposable elements. The small interfering RNAs comprise a more heterogeneous group that includes small RNAs derived from cleavage of exogenous double-stranded RNA (exo-siRNAs) or generated endogenously (endo-siRNAs).

Chemical modification has emerged as an important theme in regulation of small RNA function (for a review, see Kim et al., 2010 [3]). Internal editing has been found to occur in select miRNA precursors through the action of ADAR (*a*denosine *d*eaminase *a*cting on *R*NA) enzymes, with consequences for miRNA processing efficiency, stability, and targeting [4]–[8]. Some siRNAs generated in fly and mouse also show evidence of editing by ADARs [9], [10], but the significance of such internal editing among siRNAs is not yet known. In contrast, terminal editing through 2′-O-methylation, addition of untemplated nucleotides, or exonucleolytic trimming plays a more general role in small RNA metabolism. These terminal modifications are not unrelated. Evidence in plants and animals suggests that methylation of the 3′ terminal nucleotide protects small RNAs from polyuridylation and polyadenylation, signals that direct exonucleolytic degradation [11]–[16]. Thus, terminal methylation plays an important role in regulating small RNA turnover. Formation of the 2′-O-methyl group is catalyzed by HEN1, a methyltransferase discovered in *Arabidopsis thaliana* that is conserved across metazoa, fungi, viridiplantae, and bacteria [17]. Although plant and animal HEN1 orthologs exhibit 40–50% amino acid similarity in the conserved methyltransferase domain [18], the proteins differ in their substrate specificity. Plant HEN1 acts on small RNAs in duplex and methylates both siRNAs and miRNAs [19]–[21]. In contrast, animal HEN1 orthologs modify only single-stranded small RNAs [22]–[24], enabling methylation of small RNAs such as piRNAs, which are not derived from double-stranded RNA intermediates [25]–[29]. While animal piRNAs appear to be universally methylated [24], [26], [27], [30]–[32], animal miRNAs are generally not methylated [19], [26], [31], and the mechanisms by which animal HEN1 orthologs discriminate between substrates are not entirely clear. HEN1 orthologs that catalyze terminal methylation of small RNAs have been characterized in mouse, fish, and fly, among other organisms [15], [22]–[24], [33], yet the orthologous methyltransferase in worm [18] has yet to be investigated. With its expanded Argonaute family and diverse small RNA classes, *Caenorhabditis elegans* provides an advantage for studying HEN1 substrate specificity.

Since the discovery of the founding members of the microRNA family in *C. elegans* [1], [2], [34], many additional classes of small RNAs have been characterized. A large-scale small RNA sequencing effort revealed a class of terminally methylated 21-nucleotide RNAs with 5′ uridines [27]. These 21U RNAs were subsequently determined to represent the piRNAs of *C. elegans* based on their germline-specific expression, association with worm Piwi Argonautes PRG-1 and PRG-2, and function in transposon silencing and maintenance of temperature-dependent fertility [35]–[38]. Also found through small RNA cloning and deep sequencing were populations of 26- and 22-nucleotide RNAs with a 5′ preference for guanosine (the 26G RNAs and 22G RNAs, respectively) that constitute the endo-siRNAs of *C. elegans* [27], [39]. The 26G RNAs are primary endo-siRNAs generated in the germline to regulate spermatogenic and zygotic gene expression. They are divided into two non-overlapping subclasses named for the Argonautes that bind them: the ERGO-1 class 26G RNAs, which are generated in the maternal germline and distributed into the embryo, and the ALG-3/ALG-4 class 26G RNAs, which are specific to the male germline and required for sperm function [40]–[42]. The 22G RNAs are composed of many small RNA classes, all of which are bound by worm-specific Argonautes (Wagos). A large population of 22G RNAs are secondary endo-siRNAs whose production by RNA-dependent RNA polymerases is triggered by the activity of 21U RNAs and 26G RNAs [36], [41]–[43]; however, many other 22G RNAs are independent of these primary small RNAs [44], [45]. Secondary siRNAs serve to amplify the signal of primary small RNAs to effect robust silencing. Production of 22G secondary siRNAs is also triggered by exogenously introduced dsRNAs [43], [45]–[47], suggesting convergence of endogenous and exogenous RNAi pathways at the level of the secondary siRNA response.

Among *C. elegans* small RNAs, only 21U RNAs and 26G RNAs are known to be methylated [27], [42]; 22G RNAs triggered by either primary endo- or exo-siRNAs appear to be unmethylated [45], [46]. Although the significance of worm small RNA methylation is unknown, loss of terminal methylation has been shown to decrease stability of piRNAs in many animal models [15], [22], [24] and both endo- and exo-siRNAs in fly [22], [48]. Methylation may therefore represent an essential step in stabilization of some classes of worm small RNAs.

In this study, we characterize the *C. elegans hen1* ortholog, which has been named *henn-1* (*hen* of *n*ematode), as the name *hen-1* has already been assigned to an unrelated *C. elegans* gene. We demonstrate that HENN-1 methylates small RNAs bound by Piwi clade Argonautes: the 21U RNAs and the ERGO-1 class 26G RNAs. However, we show that 26G RNAs bound by Ago clade Argonautes ALG-3 and ALG-4 are not methylated and are therefore *henn-1*-independent. Differential methylation of 26G RNAs provides evidence for an existing model [13], [22], [23], [49], [50] wherein evolutionarily divergent Argonautes either direct or prohibit HEN1-mediated methylation of associated small RNAs. In further support of this Argonaute-dictated methylation model, we find that small RNAs are likely methylated after associating with an Argonaute: the Argonaute ERGO-1 is required for 26G RNA methylation, but methylation is not required for ERGO-1 to bind a 26G RNA.

In the *henn-1* mutant, levels of both 21U RNAs and ERGO-1 class 26G RNAs drop precipitously after their deposition into embryo, suggesting that HENN-1-mediated methylation is essential for perdurance of the maternal small RNA load during filial development. Accordingly, the *henn-1* mutant shows enhanced somatic sensitivity to exogenous RNAi, a phenotype associated with loss of ERGO-1 class 26G RNAs. Surprisingly, however, the *henn-1* mutant germline exhibits an attenuated response to RNAi, suggesting that HENN-1 may also function in the exogenous RNAi pathway. Altogether, our study supports a role for HENN-1 in diverse small RNA pathways in *C. elegans* and offers further insight into the mechanisms governing substrate discrimination for animal HEN1 orthologs.

## Results

### *C02F5.6* Encodes the *C. elegans* HEN1 Ortholog

To examine small RNA methylation in *C. elegans*, we began by characterizing *C02F5.6*, the gene previously predicted to encode the HEN1 ortholog in worm [18]. This gene, subsequently named *henn-1*, encodes a protein that exhibits significant amino acid similarity across the conserved HEN1 methyltransferase domain relative to established members of the HEN1 family (Figure S1). Although two *henn-1* gene models with differing 3′ ends have been proposed, 3′RACE and protein studies using a rabbit polyclonal antibody generated against a common N-terminal HENN-1 epitope detected only the longer isoform (Figure S2A, S2B).

To facilitate our studies of the function of HENN-1, we isolated and characterized the *henn-1(tm4477)* allele. This allele carries a deletion that encompasses *henn-1* exon four, which encodes 65% of the conserved methyltransferase domain as annotated by Kamminga et al. [15]. Sequencing of the *henn-1(tm4477)* mRNA indicates that loss of exon four activates a cryptic splice donor site in the third intron, resulting in an extended third exon that encodes a premature termination codon (Figure S2B). The *henn-1(tm4477)* mRNA is readily detected by RT-PCR but does not produce a detectable protein product (Figure S2A) or exhibit methyltransferase activity (see below), suggesting that *henn-1(tm4477)* (hereafter, *henn-1*) represents a functional null allele.

### HENN-1 Terminally Methylates and Stabilizes *C. elegans* piRNAs

**Figure 1:**
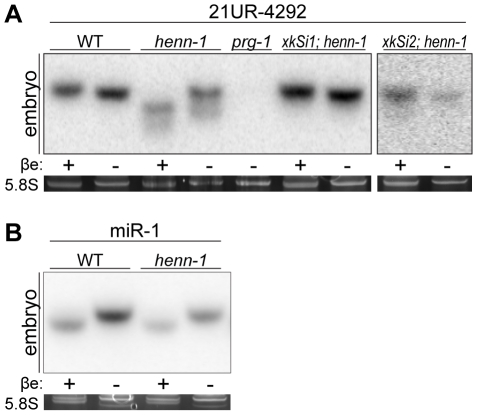
Methylation of 21U RNAs Requires *C. elegans* HEN1 Ortholog HENN-1. A) HENN-1 is required for 21U RNA methylation. Endogenous (*xkSi1*) and germline-specific (*xkSi2*) expression of *henn-1::gfp* rescue 21U RNA methylation in *henn-1(tm4477)* mutant embryo. Total embryo RNA of the indicated genotypes was β-eliminated (βe +) or control treated (βe −) and probed for piRNA 21UR-4292. *prg-1(tm872)* lacks 21U RNAs and is included as a negative control. Below, ethidium bromide staining of 5.8S rRNA is shown. Additional 21U RNA northern blots are shown in Figure S3A. B) *C. elegans* miRNAs are unmethylated. Total embryo RNA was probed for miR-1. Variable intensity of 5.8S rRNA bands in embryo indicates unequal loading.

Like piRNAs in fly [22], [23], [32], mouse [30], [31], and zebrafish [26], the *C. elegans* 21U RNAs are terminally methylated [27], but the factor catalyzing this modification has not yet been identified. To determine if 21U RNA methylation depends on *henn-1*, we assessed methylation status using the β-elimination assay [51]. A small RNA molecule whose terminal nucleotide has been 2′-O-methylated is resistant to this treatment, whereas the cis-diols of an unmodified 3′ terminal nucleotide are oxidized by sodium periodate, rendering the nucleotide susceptible to β-elimination under basic conditions. The resulting size difference can be resolved on a polyacrylamide gel to determine methylation status. All 21U RNAs examined were found to be terminally methylated in a *henn-1*-dependent manner (Figure 1A, Figure S3A), whereas a control miRNA was not methylated in either wild-type or *henn-1* mutant animals (Figure 1B). Although 21U RNAs are still detectable in the *henn-1* mutant, the abundance of the full-length species is visibly decreased for some 21U RNAs; this correlates with the appearance of putative degradation products of unmethylated, unprotected 21U RNAs. To demonstrate that loss of 21U RNA methylation in the *henn-1* mutant is specifically due to the absence of *henn-1*, we used the Mos1-mediated single copy insertion technique [52] to introduce a *henn-1::gfp* transgene driven by the promoter of the polycistronic mRNA that encodes *henn-1* (*xkSi1*) or by the germline-specific *pie-1* promoter (*xkSi2*) into the *henn-1* mutant (Figure S2C). Both endogenous and germline-specific expression of *henn-1::gfp* restore 21U RNA methylation in the *henn-1* mutant (Figure 1A).

**Figure 2:**
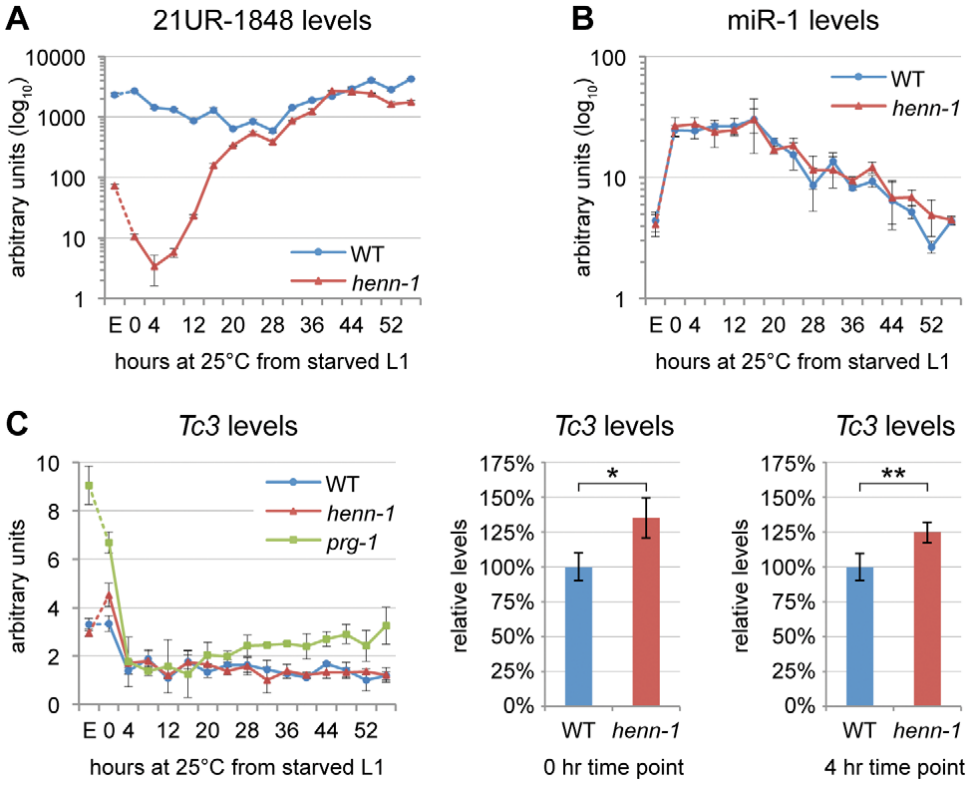
HENN-1 Stabilizes 21U RNAs. A) Loss of *henn-1* impairs 21U RNA accumulation in adult, embryo, and early larva. Levels of 21UR-1848 were assayed by Taqman qPCR in embryo and every four hours across development of wild-type and *henn-1(tm4477)* mutant animals at 25°C. Standard deviation is shown for biological triplicates. Taqman qPCR data for eight additional 21U RNAs are shown in Figure S4. B) Effects of loss of *henn-1* are restricted to its small RNA substrates. Levels of miR-1 across development were assayed by Taqman qPCR. Standard deviation is shown for biological triplicates. Additional Taqman qPCR data for miRNAs are shown in Figure S5. C) Loss of *henn-1* impairs *Tc3* transposase silencing primarily in early L1 larva. *Tc3* transposase mRNA levels were assayed by qPCR across development and normalized to mRNA levels of *eft-2*, an abundantly expressed housekeeping gene. *prg-1(tm872)* lacks 21U RNAs and is included as a positive control for *Tc3* upregulation. Significant zero and four hour time points are expanded at right (*: P = 0.0251; **: P = 0.0250, two-tailed *t*-test). Standard deviation is shown for biological triplicates. E, embryo; hr, hour.

To investigate the relationship between terminal methylation and piRNA accumulation, we used Taqman RT-qPCR to assess 21U RNA levels in wild-type and *henn-1* mutant animals across development at 25°C. Importantly, the Taqman stem-loop RT primer is capable of distinguishing between full-length and terminally degraded small RNAs [53]. For example, the *let-7e* miRNA differs from *let-7a* only in the absence of the final nucleotide and U>G substitution at the ninth nucleotide, a position likely not represented in the stem-loop Taqman primer. Absence of this final nucleotide decreases detection of *let-7e* by the *let-7a* Taqman assay by more than a thousandfold [53]. *henn-1* mutant embryo and early larva show dramatically reduced detection of female germline-enriched piRNA 21UR-1848 (Figure 2A), consistent with decreased embryonic detection for some 21U RNAs observed by northern blot (Figure 1A, Figure S3A). 21U RNA levels recover to wild-type in late larval stages, coincident with the onset of germline proliferation and de novo 21U RNA biosynthesis; however, in gravid animals at 56 hours, 21UR-1848 levels in the *henn-1* mutant have declined to less than 50% of those observed in wild-type (P = 0.0005; two-tailed *t*-test). Eight additional 21U RNAs examined show a similar pattern (Figure S4). These data suggest that *henn-1* is dispensable for piRNA biogenesis but essential for robust inheritance of piRNAs. Parallel analysis of miR-1 and several additional miRNAs across development shows that effects of loss of *henn-1* are specific to its substrates and not due to generalized small RNA dysregulation in the *henn-1* mutant (Figure 2B, Figure S5).

### HENN-1 Plays a Minor Role in piRNA–Mediated Germline Regulation

We next sought to determine the extent to which decreased abundance of piRNAs in the *henn-1* mutant compromises activity of the piRNA pathway. Unlike in fly, where many selfish genetic elements are desilenced in the absence of piRNAs [32], *C. elegans* at present has only a single established molecular readout for piRNA pathway function: increased expression of transposase mRNA from *Tc3*, a Tc1/mariner family transposon [35], [36]. Two 21U RNAs have been found to map to *Tc3*, but both map in the sense direction and thus are unlikely to act directly in *Tc3* repression via canonical RNAi mechanisms [35], [36]. Rather, 21U RNAs likely mediate their repressive effects through triggering production of secondary siRNAs, 22G RNAs, that engage worm-specific Argonautes (Wagos) to effect *Tc3* gene silencing [36], [45]. We therefore identified a 22G RNA that shows complete antisense complementarity to *Tc3* and can be classified as a Wago-dependent, 21U RNA-dependent secondary siRNA based on its total depletion both in the MAGO12 mutant, which lacks all Wagos, and in the *prg-1(n4357); prg-2(n4358)* double mutant, which lacks piRNAs [36], [45]. Levels of this 22G RNA in the *henn-1* mutant are reduced by 44% in embryo but not significantly altered in hatched L1 larva (Figure S6A). This suggests that the low embryonic and early larval levels of 21U RNAs in the *henn-1* mutant are still sufficient to trigger production of secondary siRNAs, although to a lesser degree than in wild-type.

Consistent with the modest effect of loss of *henn-1* on accumulation of piRNA-triggered secondary siRNAs, *henn-1* mutant animals exhibit only a small increase (35% in starved L1 larva, 25% in L1 larva fed for 4 hours at 25°C) in *Tc3* transposase mRNA levels relative to wild-type (Figure 2C). This is not unexpected due to the poor coincidence of the time intervals corresponding to piRNA dysregulation in the *henn-1* mutant and *Tc3* sensitivity to 21U RNAs; the *henn-1* mutant shows the greatest disparity in piRNA levels in early larval development, whereas *Tc3* levels are most sensitive to piRNAs in germline and embryo (Figure 2A, 2C). These findings suggest that HENN-1 is not strictly required for piRNA target repression, but contributes to robust silencing of *Tc3*.

In addition to *Tc3* dysregulation, loss of *prg-1* also results in a temperature-sensitive sterility phenotype [38], [43]. To determine if the *henn-1* mutant also exhibits a fertility defect, we assessed fertility at 20°C and 25°C. At 20°C, brood size of the *henn-1* mutant does not differ significantly from that of wild-type. In contrast, *henn-1* mutant animals maintained at 25°C exhibit a 25% decrease in brood size relative to wild-type (P = 0.0059; two-tailed *t*-test) that can be rescued by germline expression of *henn-1::gfp* from the *xkSi2* transgene (Figure S7). The impaired fertility of the *henn-1* mutant is consistent with abnormal fertility phenotypes associated with loss of HEN1 methyltransferase activity in other animals. Loss of *HEN1* in *Tetrahymena thermophila* depletes Piwi-interacting RNAs called scan RNAs, impairing DNA elimination and, consequently, the viability of progeny [24]. The zebrafish *hen1* mutant fails to maintain a female germline, resulting in an exclusively male population [15]. Nevertheless, we cannot conclude that the temperature-sensitive fertility defect of the *henn-1* mutant is due exclusively to compromise of the 21U RNA pathway.

### ERGO-1 and ALG-3/ALG-4 Class 26G RNAs Are Differentially Methylated by HENN-1

**Figure 3:**
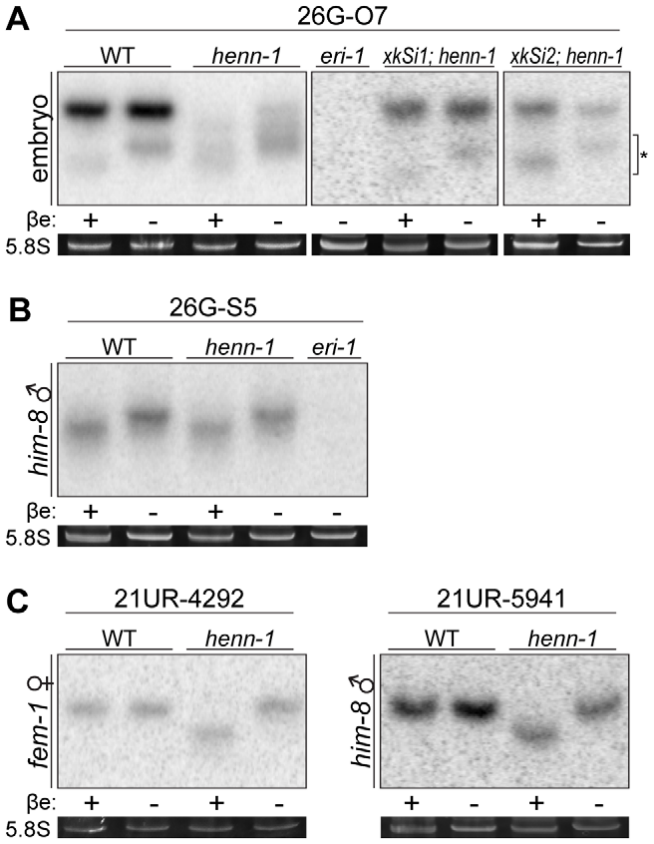
HENN-1 Selectively Methylates ERGO-1 Class 26G RNAs in an ERGO-1–Dependent Manner. A) HENN-1 is required for ERGO-1 class 26G RNA methylation and stability. Total β-eliminated (βe +) or control treated (βe −) embryo RNA of the indicated genotypes was probed for ERGO-1 class 26G RNA 26G-O7. *eri-1(mg366)* lacks 26G RNAs and is included as a negative control. Asterisk indicates signal corresponding to cross-hybridization with unmethylated 22G RNAs. Below, ethidium bromide staining of 5.8S rRNA. Additional ERGO-1 class 26G RNA northern blots are shown in Figure S3B. B) ALG-3/ALG-4 class 26G RNAs are unmethylated. Total *him-8(e1489)* male RNA was probed for ALG-3/ALG-4 class 26G RNA 26G-S5. An additional ALG-3/ALG-4 class 26G RNA northern blot is shown in Figure S3C. C) 21U RNAs are methylated in a HENN-1-dependent manner in both female and male germlines. Total RNA of the indicated genotypes from *fem-1(hc17)* female or *him-8(e1489)* male was probed for female germline-enriched piRNA 21UR-4292 or male germline-enriched piRNA 21UR-5941, respectively.

26G RNAs were reported to be methylated in the first *C. elegans* small RNA deep sequencing study [27]. Subsequent studies concluded that the species assessed was an ERGO-1 class 26G RNA [40]. Consistent with these data, we found that ERGO-1 class 26G RNAs, found in female germline and embryo, are methylated. As was the case for piRNAs, this methylation occurs in a *henn-1*-dependent manner (Figure 3A, Figure S3B). Surprisingly, however, ALG-3/ALG-4 class 26G RNAs, specific to the male germline, showed no evidence of methylation even in wild-type animals (Figure 3B, Figure S3C). One potential explanation for this observation would be that female germline small RNAs are universally methylated, whereas male germline small RNAs are not. To explore this possibility, we assessed 21U RNAs in male and female germlines. Both were methylated (Figure 3C), indicating that differential 26G RNA methylation cannot be explained simply by a lack of methyltransferase functionality in the male germline.

**Figure 4:**
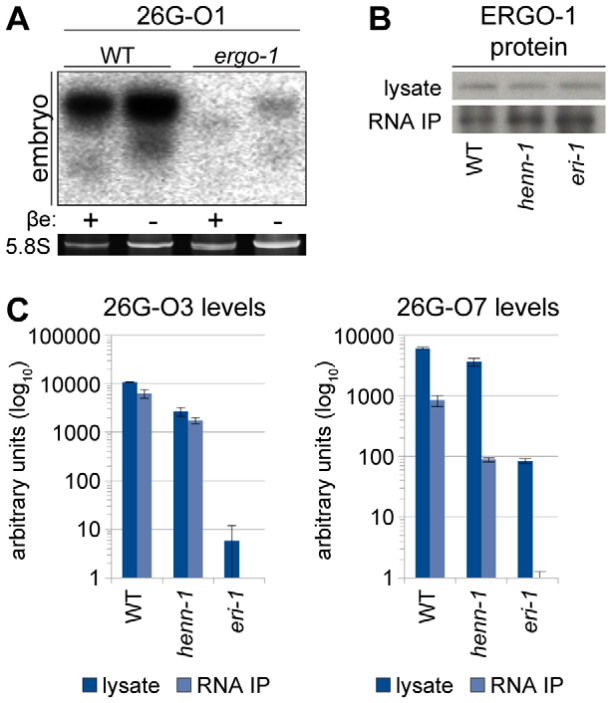
ERGO-1 Is Required for Methylation of 26G RNAs. A) ERGO-1 class 26G RNA 26G-O1 is unmethylated in the absence of ERGO-1. Total embryo wild-type (5 µg) or *ergo-1(tm1860)* (10 µg) β-eliminated (βe +) or control treated (βe −) RNA was probed for 26G-O1. B) Anti-ERGO-1 rabbit polyclonal antibody immunoprecipitates ERGO-1 complexes. ERGO-1 complexes were immunopurified from lysates of equalized protein concentration extracted from wild-type, *henn-1(tm4477)* mutant, or *eri-1(mg366)* mutant embryo. Aliquots of lysates and immunoprecipitates (RNA IP) were probed with anti-ERGO-1 antibody. *ergo-1(tm1860)* mutant lysate was run in parallel to ensure specificity of ERGO-1 detection (data not shown). C) ERGO-1 binds methylated and unmethylated 26G RNAs. Taqman RT-qPCR for the indicated ERGO-1 class 26G RNAs was performed on samples described in B. The *eri-1(mg366)* mutant lacks 26G RNAs and serves as a negative control to demonstrate specificity of 26G RNA detection by Taqman assay. Standard deviation is shown for technical duplicates. Results are representative of two independent RNA immunoprecipitation experiments.

Because the two classes of 26G RNAs bind unique Argonautes in male and female germlines, we hypothesized that the Argonaute ERGO-1 might direct methylation of 26G RNAs. To address this question, we sought to assess methylation of an ERGO-1 class 26G RNA in the absence of ERGO-1. As 26G RNAs are dramatically depleted in the absence of their respective Argonautes [40], we queried published wild-type and *ergo-1(tm1860)* gravid adult deep sequencing libraries [42] to identify an ERGO-1 class 26G RNA that still accumulates to levels sufficient for visualization by northern blotting in the *ergo-1(tm1860)* mutant. 26G-O1, an extremely abundant ERGO-1 class 26G RNA, is present at roughly 0.5% wild-type levels in the *ergo-1(tm1860)* mutant, but still abundant enough to detect by northern blotting. Consistent with our hypothesis that ERGO-1 is required for 26G RNA methylation, we found that 26G-O1 is unmethylated in the *ergo-1(tm1860)* mutant embryo (Figure 4A). We next asked the converse question: Is 26G RNA methylation required for association with ERGO-1? We immunopurified ERGO-1 complexes from wild-type and *henn-1* mutant embryo lysates (Figure 4B) and extracted RNA. In both wild-type and *henn-1* mutant samples, ERGO-1 class 26G RNAs are readily detected (Figure 4C), indicating that ERGO-1 effectively binds both methylated and unmethylated 26G RNAs. Taken together, these data suggest that 26G RNAs bind ERGO-1 and are subsequently methylated by HENN-1.

**Figure 5:**
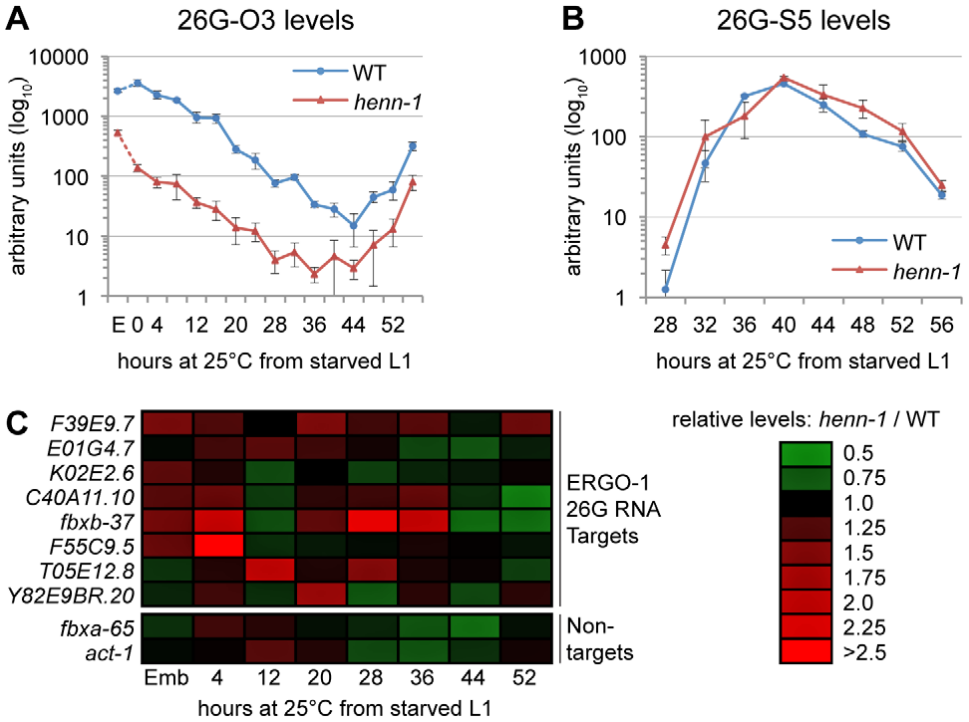
HEN1 Stabilizes ERGO-1 Class, but Not ALG-3/ALG-4 Class, 26G RNAs. A) Loss of *henn-1* impairs ERGO-1 class 26G RNA accumulation at all stages. Levels of ERGO-1 class 26G RNA 26G-O3 were assayed by Taqman qPCR across development of wild-type and *henn-1(tm4477)* mutant animals at 25°C. Standard deviation is shown for biological triplicates. Taqman qPCR data for seven additional ERGO-1 class 26G RNAs are shown in Figure S8. B) ALG-3/ALG-4 class 26G RNAs are *henn-1*-independent. Levels of ALG-3/ALG-4 class 26G RNA 26G-S5 were assayed across the period of development in which ALG-3/ALG-4 class 26G RNAs are readily detectable. Standard deviation is shown for biological triplicates. Taqman qPCR data for two additional ALG-3/ALG-4 class 26G RNAs are shown in Figure S9. C) Loss of *henn-1* may result in modest, sporadic defects in ERGO-1 class 26G RNA target silencing. Levels of eight target and two non-target mRNAs were assayed across development of wild-type and *henn-1(tm4477)* mutant animals at 25°C and normalized to *eft-2*. Expression in the *henn-1(tm4477)* mutant relative to wild-type is represented according to the red-green color scheme indicated in the right panel. Raw data is shown in Figure S10. E, embryo.

To test whether HENN-1-mediated methylation is required to maintain levels of all substrate small RNAs, we assessed ERGO-1 class 26G RNAs for defects in accumulation in the *henn-1* mutant. Loss of *henn-1* has more severe consequences for this class of small RNAs than are observed for 21U RNAs: ERGO-1 class 26G RNA 26G-O3 fails to accumulate to wild-type levels at any stage of development, although the disparity is less pronounced in adulthood, during peak 26G RNA biogenesis (Figure 5A). For comparison, we assayed levels of ALG-3/ALG-4 class 26G RNA 26G-S5 across the developmental window during which it is readily detected by Taqman RT-qPCR. Levels of 26G-S5 are similar in the *henn-1* mutant relative to wild-type (Figure 5B), consistent with the idea that HENN-1 is required for accumulation of ERGO-1 class 26G RNAs but dispensable for that of ALG-3/ALG-4 class 26G RNAs. Analysis of seven additional ERGO-1 class 26G RNAs and two additional ALG-3/ALG-4 class 26G RNAs corroborated these observations (Figures S8, S9).

### HENN-1 Contributes Minimally to ERGO-1 Class 26G RNA Target Silencing

To determine the effect of loss of *henn-1* on the silencing of ERGO-1 class 26G RNA targets, we assayed levels of a panel of mRNAs targeted by ERGO-1 class 26G RNAs for desilencing in *henn-1* mutant animals. During time points at which ERGO-1 class 26G RNAs are abundant, only modest upregulation of some, but not all, targets was detected; furthermore, no single target shows consistent desilencing in the *henn-1* mutant (Figure 5C, Figure S10A). This is not unexpected, however, as the targets themselves vary in both expression and sensitivity to small RNA-mediated silencing across development [40]. To determine the specificity of this effect, two non-targets were examined in parallel. The maximal upregulation for either non-target does not exceed the maximal upregulation observed for any target, suggesting that the upregulation of ERGO-1 class 26G RNA targets in the *henn-1* mutant may be a consequence of 26G RNA depletion (Figure 5C, Figure S10B). This connection is supported by our observation that a Wago-dependent and ERGO-1 class 26G RNA-dependent secondary siRNA that presumably enhances target silencing also shows defects in accumulation in embryo (Figure S6B). The effect is modest, indicating that, as observed for the piRNA pathway, the depleted pool of ERGO-1 class 26G RNAs in the *henn-1* mutant is still sufficient for triggering fairly robust production of secondary siRNAs. Nevertheless, in an accompanying manuscript, Montgomery et al. observe that HENN-1 is required for silencing activity of a similar secondary siRNA upon a sensor transgene [54], suggesting that this pathway may indeed be compromised by loss of *henn-1*.

### The Soma of the *henn-1* Mutant Exhibits Enhanced Sensitivity to Exogenous RNAi

ALG-3/ALG-4 class 26G RNAs are restricted to the male germline, and their mRNA targets are enriched for genes involved in spermatogenesis [40]. Accordingly, loss of ALG-3/ALG-4 class 26G RNAs results in male-associated sterility at non-permissive temperatures due to defects in sperm activation that are thought to arise from target dysregulation [41]. ERGO-1 class 26G RNAs, in contrast, are dispensable for fertility and target mostly poorly conserved and incompletely annotated genes, many of which reside in duplicated regions of the genome [42]. It is therefore not unexpected that the *ergo-1(tm1860)* mutant, which lacks ERGO-1 class 26G RNAs, exhibits no overt phenotypes that can be traced to target dysregulation. Rather, the *ergo-1(tm1860)* mutant exhibits an *e*nhanced *R*NA*i* sensitivity (Eri) phenotype that is attributed to effects of loss of the ERGO-1-dependent small RNAs themselves; presumably, depletion of ERGO-1 class 26G RNAs and dependent secondary siRNAs liberates limiting RNAi factors shared between the endogenous and exogenous RNAi pathways [43], [55], [56].

**Figure 6:**
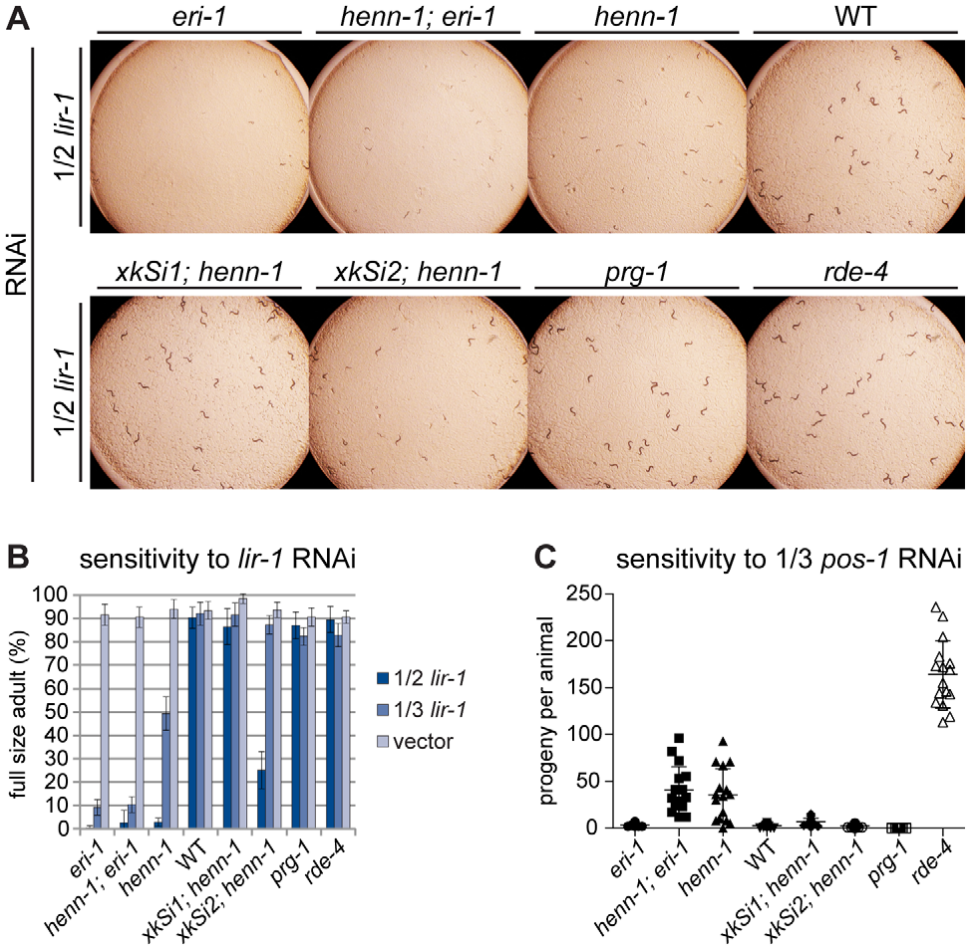
The *henn-1* Mutant Exhibits Opposite RNAi Sensitivity Phenotypes in Soma and Germline. A) *henn-1(tm4477)* mutant animals exhibit mildly enhanced somatic RNAi. Animals of the indicated genotype were plated as L1 larvae on *lir-1* feeding RNAi diluted 1∶1 with empty vector (1/2 strength) and grown for 70 hours at 20°C. Data is quantified in part B. RNAi sensitivity data for knockdown of two additional somatic transcripts are shown in Figure S11. B) Endogenous expression of *henn-1::gfp* from *xkSi1* rescues somatic RNAi sensitivity. Percent of animals reaching full size on *lir-1* feeding RNAi of the indicated strength at 70 hours is plotted. N = 8 plates of >50 animals per strain. Standard deviation is shown. C) *henn-1(tm4477)* mutant animals exhibit defective germline RNAi. Brood size of animals plated at 20°C as L1 larvae on *pos-1* feeding RNAi diluted 1∶2 with empty vector is plotted. N≥13 animals per strain. Mean and standard deviation are shown. RNAi sensitivity data for knockdown of four additional germline transcripts are shown in Figure S12. Alleles used in this figure: *eri-1(mg366)*, *prg-1(tm872)*, *rde-4(ne301)*.

To determine whether loss of *henn-1* depletes ERGO-1 class 26G RNAs sufficiently to produce an Eri phenotype, as observed in the *ergo-1* mutant, we subjected L1 larvae from a panel of strains to feeding RNAi targeting various genes in the soma or germline. In order to expose subtle differences in RNAi sensitivity, we modulated the degree of knockdown, attenuating the dose of dsRNA trigger by diluting the bacterial RNAi clone with a bacterial clone harboring empty vector. RNAi of the somatic gene *lir-1* causes larval arrest and lethality in wild-type animals at full strength, but dilution 1∶1 with empty vector largely eliminates the effect. In contrast, the *eri-1(mg366)* mutant, which lacks 26G RNAs, is affected severely by even dilute *lir-1* RNAi. The *henn-1* mutant also shows dramatically increased sensitivity to *lir-1* feeding RNAi relative to wild-type (Figure 6A, 6B). A *henn-1; eri-1* double mutant, however, shows RNAi sensitivity that is virtually identical to that of the single *eri-1* mutant, suggesting that the Eri phenotype of each allele likely stems from the same defect, namely, loss of ERGO-1 class 26G RNAs. While the somatic Eri phenotype of the *henn-1* mutant shows partial rescue by the germline-specific *henn-1::gfp* transgene *xkSi2*, *henn-1::gfp* expression under the native promoter from transgene *xkSi1* rescues wild type RNAi sensitivity completely in the *henn-1* mutant (Figure 6B). These findings suggest that loss of *henn-1* in both germline and soma contributes to the Eri phenotype of the *henn-1* mutant. The *henn-1* mutant exhibits a similar somatic Eri response to RNAi of *dpy-13* and *lin-29* (Figure S11).

### The Germline of the *henn-1* Mutant Exhibits Decreased Sensitivity to Exogenous RNAi

While the somatic Eri phenotype of the *henn-1* mutant was expected, knockdown of genes required for germline development or embryogenesis revealed that, incongruously, the *henn-1* mutant maternal germline exhibits an *R*NAi *de*fective (Rde) phenotype. Animals subjected to *pos-1* RNAi lay dead embryos because maternally loaded *pos-1* mRNA is required for specifying cell fate of many tissues during embryonic development [57]. On *pos-1* RNAi diluted 1∶2 with empty vector (1/3 strength), knockdown in wild-type animals is still sufficiently robust to reduce average brood size to fewer than five offspring per animal. *henn-1* mutant animals at this dilution, however, produce an average brood greater than tenfold that of wild-type, suggesting that loss of *henn-1* confers resistance to RNAi-mediated knockdown of this maternally deposited mRNA (Figure 6C). A lesser but statistically significant effect was observed for RNAi of the germline-expressed transcripts *par-1*, *par-2*, *pie-1*, and *glp-1* (Figure S12). Sensitivity to *pos-1* RNAi is effectively rescued by either endogenous or germline-specific expression of *henn-1::gfp*, likely due to the fact that both transgenes are expressed in germline.

### HENN-1 Is Expressed in Both Germline and Soma

**Figure 7:**
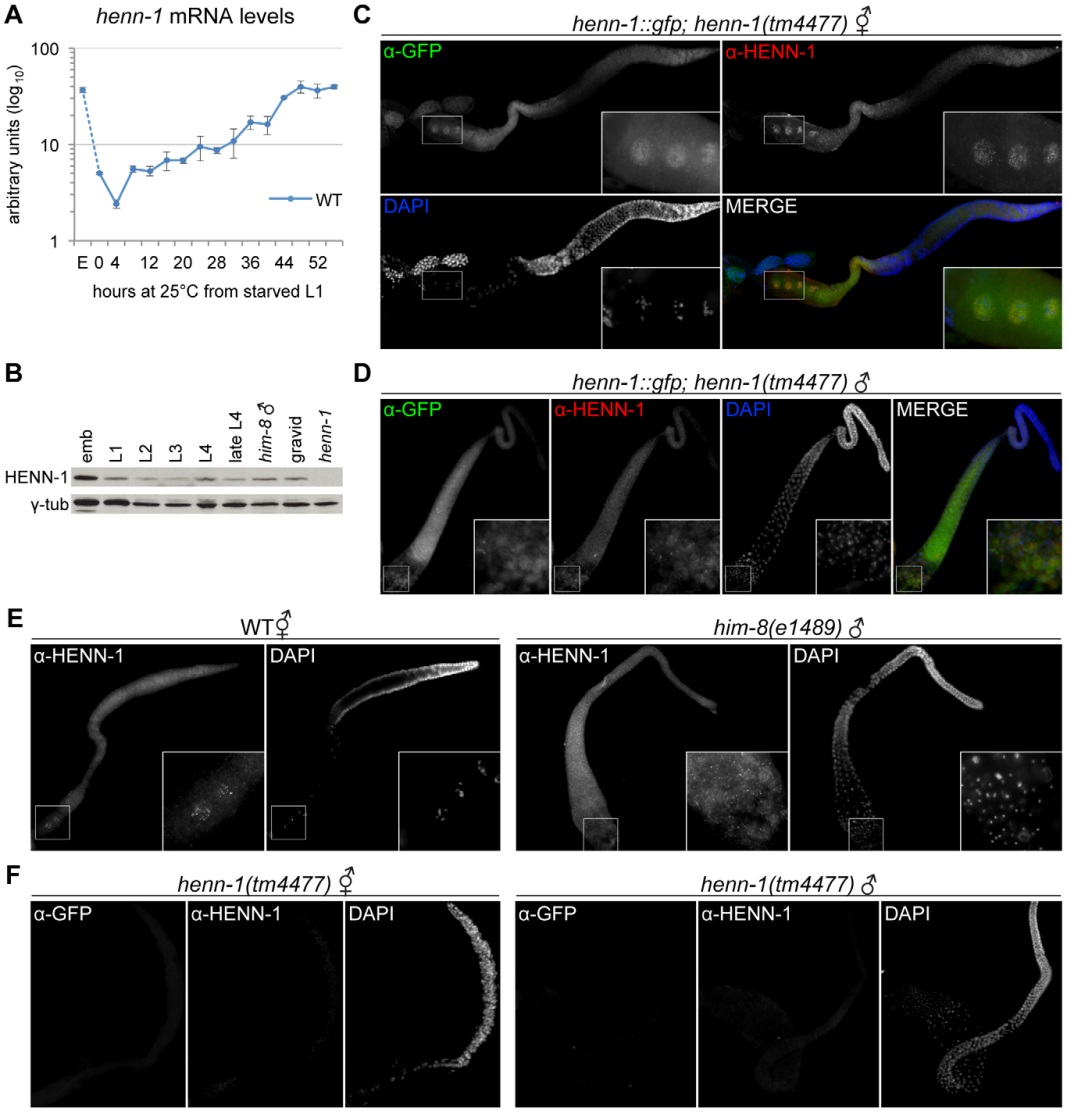
HENN-1 Is Broadly Expressed in *C. elegans* Germline. A) The *henn-1* mRNA expression profile is consistent with germline enrichment. Levels of *henn-1* mRNA were assayed throughout development and normalized to *eft-2* mRNA. Standard deviation is shown for biological triplicates. Non-normalized levels are shown in Figure S13A. B) HENN-1 is detected at all stages of development and in male. Lysates from animals of the indicated stages were probed with anti-HENN-1 rabbit polyclonal antibody. C) HENN-1 is abundant in hermaphrodite proximal germline and enriched in proximal oocyte nucleoplasm (inset). Extruded gonads of *xkSi1; henn-1(tm4477)* adult hermaphrodites were stained with anti-GFP mouse monoclonal and anti-HENN-1 rabbit polyclonal antibodies. D) HENN-1 is detectable in male proximal and distal gonad, with enrichment in residual bodies during spermatid maturation (inset). Extruded gonads of *xkSi1; henn-1(tm4477)* adult males were stained with anti-GFP and anti-HENN-1 antibodies. E) Expression of endogenous HENN-1 mirrors expression of HENN-1::GFP from transgene *xkSi1*. Extruded gonads of wild-type animals were stained with anti-HENN-1 antibody. F) Detection of HENN-1 proteins by immunostaining is specific. Extruded gonads of *henn-1(tm4477)* mutant animals were stained with anti-GFP and anti-HENN-1 antibodies. E, embryo.

HEN1 orthologs appear to be restricted to the germline in vertebrates [15], [33]; however, we observe phenotypes in both the germline and soma of the *henn-1* mutant that suggest broader activity. To investigate expression of HENN-1 in *C. elegans*, we assessed *henn-1* mRNA and protein levels throughout development. *henn-1* mRNA levels are lowest in young larva and increase as the germline proliferates, peaking in gravid adult (Figure 7A, Figure S13A). Germline-deficient *glp-4(bn2)* adult hermaphrodites show approximately a 50% reduction in *henn-1* mRNA levels relative to wild-type (Figure S13B), indicating that *henn-1* mRNA is expressed in both germline and soma. Embryonic levels of *henn-1* are high but decrease rapidly; this pattern suggests that, unlike in zebrafish [15], *henn-1* mRNA may be maternally deposited into the embryo. HENN-1 protein is detectable throughout development and in both hermaphrodite and male adults (Figure 7B).

We next assessed the distribution of HENN-1::GFP fusion protein expressed from *xkSi1*, the rescuing *henn-1::gfp* transgene driven by the endogenous promoter, in the *henn-1* mutant background. Although single copy transgene expression levels are too low for direct visualization by fluorescence microscopy, HENN-1::GFP is readily detected using a mouse monoclonal anti-GFP antibody. Whole-mount immunostaining of transgenic L4 larvae reveals that HENN-1::GFP is expressed broadly in diverse somatic tissues and germline (Figure S13C). Non-transgenic larvae show no signal, indicating that detection of HENN-1::GFP is specific. In extruded gonads of *xkSi1; henn-1* hermaphrodites, HENN-1::GFP is detected throughout the germline. Notably, the proximal oocytes show cytoplasmic and intense nucleoplasmic HENN-1::GFP expression (Figure 7C). Although nucleoplasmic enrichment is lost following fertilization, HENN-1::GFP is also abundant in embryo, with ubiquitous expression prior to gastrulation (Figure S13D). HENN-1::GFP is also expressed throughout the germline of *xkSi1; henn-1* males (Figure 7D). During sperm maturation, we detect enrichment of HENN-1::GFP in residual bodies, but we cannot definitively conclude that it is excluded from sperm (Figure 7D, inset). In wild-type animals, studies of endogenous HENN-1 using the rabbit polyclonal antibody generated against an N-terminal HENN-1 epitope corroborate the above findings, although the signal is more difficult to detect (Figure 7E). Staining in the *henn-1* mutant yields no signal for anti-GFP and anti-HENN-1 antibodies (Figure 7F); this demonstrates that detection of transgenic and endogenous HENN-1 proteins is specific. Together, these data define an expression pattern consistent with a role for HENN-1 in modifying small RNAs in both male and female germlines as well as in soma.

The 21U RNAs and 26G RNAs appear to be significantly stable only in the presence of their respective Argonaute proteins [35], [36], [40]; accordingly, the localization patterns of the Argonaute proteins reflect the distribution of the different classes of small RNAs. We therefore wanted to compare the expression patterns of HENN-1 and the 26G RNA-binding Argonautes to determine whether the small RNA substrate specificity of HENN-1 could be explained by differential access to Argonaute-bound small RNAs. ERGO-1, which binds methylated 26G RNAs, is abundant in embryo [42], and its transcript is enriched during oogenesis [58], but its localization has not yet been reported. We assessed the staining pattern of ERGO-1 in hermaphrodite gonad and embryo using a polyclonal antibody generated against a C-terminal ERGO-1 epitope. ERGO-1 expression in the hermaphrodite germline begins at pachytene exit and persists in embryo (Figure S13D, S13E). ERGO-1 shows cytoplasmic enrichment both in germline and embryo, suggesting that the cytoplasmic pool of HENN-1 may act in methylating 26G RNAs bound by ERGO-1. This interaction may, however, be transient, as we were unable to identify HENN-1 by mass spectrometry of immunopurified ERGO-1 complexes, nor could we detect ERGO-1 in immunopurified HENN-1::GFP complexes by western blot (data not shown). Notably, both HENN-1 and ERGO-1 remain abundant in early embryo (Figure S13D). This is consistent with the proposed existence of a somatic endo-siRNA pathway that promotes continued biosynthesis of ERGO-1 class 26G RNAs after fertilization [59].

We next assessed co-localization of HENN-1 and ALG-3. ALG-3 and its close paralog, ALG-4, bind unmethylated 26G RNAs, and their transcripts are enriched during spermatogenesis [58]. In the male gonad, a rescuing *gfp::alg-3* transgene was reported to express in the proximal male germline, with localization to P granules beginning at late pachytene [41]. During sperm maturation, GFP::ALG-3 is relegated to residual bodies. Dual immunostaining of GFP::ALG-3 and endogenous HENN-1 demonstrates a large region of overlap (Figure S13F), but HENN-1 does not appear to localize to P granules. This does not explain why ALG-3/ALG-4 class 26G RNAs are not methylated, because it is likely that HENN-1 can access P granules transiently: PRG-1 localizes predominantly to P granules [35], [37], and the PRG-1-bound piRNAs are methylated. This is in contrast to zebrafish Hen1, which carries a poorly conserved C-terminal domain (Figure S1) that directs localization of Hen1 to nuage, perinuclear granules similar to *C. elegans* P granules, to methylate piRNAs [15].

## Discussion

### Differential 26G RNA Methylation Supports an Argonaute-Dictated Methylation Model

We have shown that HENN-1 is essential for methylating select classes of *C. elegans* small RNAs, namely, 21U RNAs and ERGO-1 class 26G RNAs. As is the case in other animals, small RNAs in *C. elegans* that associate with Piwi clade Argonautes require HENN-1 for maintenance of wild-type levels. Ago clade Argonaute-associated microRNAs and ALG-3/ALG-4 class 26G RNAs, in contrast, are HENN-1-independent (Figure S14A). It has been proposed that spatial and temporal regulation of HEN1 ortholog expression may contribute to small RNA substrate specificity in metazoans [24]. However, our immunostaining studies indicate that HENN-1 is coexpressed in the same tissues and subcellular compartments as Argonautes ERGO-1, PRG-1, and ALG-3 and their respective small RNAs (Figure 7, Figure S13). Therefore, differences in gross sub-cellular localization cannot explain the failure of ALG-3/ALG-4 class 26G RNAs to be methylated. Furthermore, although the two subclasses of 26G RNAs are generated in different germlines from non-overlapping targets, their sequences exhibit no obvious distinguishing characteristics that might account for their non-uniform methylation status.

One model of small RNA methylation posits that animal HEN1 orthologs only methylate small RNAs bound by Argonautes [15], [22]–[24], [49]. In support of this, work in fly shows that siRNA methylation requires assembly of DmAgo2 RISC [22], [50], and in vitro studies using lysate from a silkworm ovary-derived cell line show that methylation of synthetic RNA only occurs after the longer substrate is bound by a Piwi protein and trimmed to piRNA size [60]. This model predicts that all 26G RNAs are bound as unmethylated species by either ERGO-1 in the female germline or ALG-3/ALG-4 in the male germline and subsequently methylated or not, respectively. This is consistent with our findings in vivo that ERGO-1 is required for methylation of 26G RNAs (Figure 4A) and associates with 26G RNAs of either methylation status (Figure 4C). It has been further proposed that the identity of the Argonaute determines whether bound small RNAs are methylated [22], [23], [49], [50]. An elegant illustration of this is provided by fly miR-277, which associates with both Ago1, the canonical fly miRNA Argonaute, and Ago2, which binds methylated siRNAs [61]. The miR-277 pool contains both methylated and unmethylated species. Depletion of Ago2 in cell culture results in loss of methylated miR-277, whereas Ago1 depletion results in a completely methylated miR-277 population [22]. Similarly, fly hairpin derived hp-esiRNAs sort into Ago1 and Ago2, but accumulate mainly in Ago2 because only hp-esiRNAs bound by Ago2 are methylated and therefore protected against degradation triggered by their extensive target complementarity [50]. In *C. elegans*, the model of Argonaute-dictated methylation can be invoked to explain the disparate methylation of the 26G RNAs: in the male germline, only ALG-3/ALG-4 are expressed, resulting in an unmethylated male 26G RNA population, whereas exclusive expression of ERGO-1 in the female germline and embryo directs methylation of female and zygotic 26G RNAs. This raises the intriguing possibility that selective expression of Argonautes that permit or prevent methylation could represent a new mechanism for differentially regulating small RNA turnover.

It is important to note that our results do not definitively exclude an alternative model wherein 26G RNAs are methylated prior to association with Argonautes and subsequently bound by ALG-3/ALG-4 only if unmethylated or by ERGO-1 only if methylated. In this model, HEN1 would methylate 26G RNAs in both germlines, but degradation of labile unbound siRNAs would result in a purely unmethylated or methylated population of 26G RNAs in male and female germlines, respectively. Because 26G RNAs assessed in embryo are fully methylated (Figure 3A, Figure S3B), such a mechanism would require that ERGO-1 exhibit very unfavorable kinetics for association with unmethylated small RNAs. We do not find this to be the case, as ERGO-1 binds some 26G RNAs with similar efficiency when methylated and unmethylated (Figure 4C). Our data therefore provide stronger evidence for a model of Argonaute-dictated methylation of small RNAs.

### Possible Advantages for Selective Methylation of Small RNAs

Differential germline expression of Argonautes could have evolved in *C. elegans* because of advantages conferred by selective stabilization of female germline 26G RNAs. Unlike ALG-3/ALG-4 class 26G RNAs, which appear to function exclusively during sperm development [40], [41], ERGO-1 class 26G RNAs exert much of their influence during embryonic and larval development, well beyond initiation of their biogenesis in the hermaphrodite germline [40]. Accordingly, their targets are depleted of germline-enriched genes [40], [59]. The oocyte contributes the vast majority of the initial zygotic cellular contents; therefore, methylation of 26G RNAs originating in the female germline may ensure robust inheritance and perdurance of primary small RNAs. Methylation of 26G RNAs in the male germline would likely not significantly increase their representation in sperm or zygote, as ALG-3/ALG-4 are relegated to residual bodies during spermatogenesis and exert effects in mature sperm only indirectly through dependent secondary 22G RNAs [41]. Nonetheless, it would be interesting to express ERGO-1 ectopically in sperm and determine whether ALG-3/ALG-4 class small RNAs are methylated. Such a strategy may reveal unexpected consequences related to inappropriate methylation and stabilization of ALG-3/ALG-4 class 26G RNAs.

### Role of HENN-1 in the Balance between Endo– and Exo–RNAi

In the absence of *henn-1*, we show that response to RNAi-mediated knockdown is enhanced for somatic genes (Figure 6A and 6B, Figure S11). This is likely due to destabilization of ERGO-1 class 26G RNAs in the *henn-1* mutant, which reduces competition with primary exo-siRNAs for stimulating secondary siRNA activity mediated by somatic Argonautes such as SAGO-1 and SAGO-2 [43], [55]. While germline-specific expression of *henn-1::gfp* only partially rescues this somatic Eri phenotype, *henn-1* mutant animals rescued with an endogenous *henn-1::gfp* transgene, which drives both somatic and germline expression, show wild-type RNAi sensitivity. Under the model of competing endo- and exo-RNAi pathways, this suggests that HENN-1-mediated methylation of ERGO-1 class 26G RNAs in the germline alone cannot maintain small RNA levels sufficient to sequester an appropriate proportion of the limiting RNAi factors. It is possible that ERGO-1 class 26G RNA biogenesis continues in embryo and larva, as previously suggested [59], and that high concentrations of HENN-1 are necessary for continued stabilization of these small RNAs. Such a model would be consistent with our characterization of the distributions of HENN-1 and ERGO-1, both of which are still detected in abundance in developing embryo (Figure S13D, S13E).

While the majority of the phenotypes observed in the *henn-1* mutant can be attributed to destabilization of endogenous small RNA substrates, the germline Rde phenotype suggests a role for HENN-1 in exogenous RNAi. It is unclear why HENN-1 is dispensable for robust exogenous RNAi in the soma but required in the germline. While this may be an indirect effect, as suggested in concurrent work by Kamminga et al. [62], one possible explanation is that HENN-1 stabilizes primary exo-siRNAs or dependent 22G secondary siRNAs. There is support in fly for methylation of exo-siRNAs and transgenic hairpin-derived siRNAs [22], [63], but this has not yet been demonstrated in *C. elegans*. 22G RNAs triggered by primary exo-siRNAs appear not to be methylated [47], consistent with our and others' observations that Wago-dependent 22G RNAs from diverse endogenous sources are unmethylated (Figure 3A, Figure S3B, and [45]). The methylation status of worm primary exo-siRNAs has not been definitively established, although a 22-nucleotide siRNA generated from a transgene encoding a perfect hairpin was not found to be methylated [46].

### Structural Differences in Ago and Piwi Clade Argonautes May Dictate HEN1 Substrate Specificity

All Argonautes contain two signature domains, PAZ and Piwi [64]. The Piwi domain, unique to Argonautes, adopts an RNase H-like configuration and serves as the catalytic core of RISC [65], [66]. The PAZ domain recognizes and anchors the 3′ end of the small RNA [67], [68]. Comparison of Piwi and Ago clade Argonautes reveals that Piwi proteins contain a small insertion in their PAZ domains in a loop connecting two β strands [69]. Crystal structures of a human Piwi Argonaute PAZ domain suggest that this insertion results in the formation of a more spacious binding pocket capable of accommodating the 2′-O-methyl group of a piRNA. Interactions between the methyl group and hydrophobic residues lining the pocket confer a threefold to sixfold higher binding affinity for 2′-O-methyl than 2′-OH [69]. In *C. elegans*, only PRG-1/PRG-2 and ERGO-1 show evidence of a PAZ domain insertion (Figure S14B), consistent with their designation as Piwi clade Argonautes and association with methylated small RNAs.

In spite of their shared classification, ERGO-1 exhibits far less homology than PRG-1/PRG-2 to mammalian and insect Piwi proteins (Figure S14A) [43]. Similarly, among worm, fly, and human Argonautes, DmAgo2 and *C. elegans* Argonaute RDE-1 are among the most divergent members of their clades [43]. In fact, so divergent is RDE-1 that its cladistics are ambiguous, with our and other published alignments variably assigning it to each of the three clades (Figure S14A and [43], [70]). Both DmAgo2 and RDE-1 bind exo-siRNAs, although only the former has been shown to permit methylation [22]. Interestingly, both lack the insertion found in Piwi Argonaute PAZ domains (Figure S14B). The absence of this insertion in DmAgo2 suggests that it is not required for association with methylated small RNAs, raising the possibility that RDE-1 too may permit methylation of associated small RNAs. If HENN-1 does not methylate RDE-1-bound small RNAs, it is unclear what specific role HENN-1 plays in exo-RNAi in the germline. Nevertheless, its dual functions in endogenous and exogenous RNAi place HENN-1 in the company of DCR-1 and the Wago proteins at the intersection between these two RNAi pathways.

## Materials and Methods

### *C. elegans* Strains

*C. elegans* were maintained according to standard procedures. The Bristol strain N2 was used as the standard wild-type strain. The alleles used in this study, listed by chromosome, are: unmapped: *neIs23[unc-119(+) GFP::ALG-3]*; LGI: *glp-4(bn2)*, *prg-1(tm872)*; LGII: *xkSi1[PC30A5.3::henn-1::gfp::henn-1 3′UTR cb-unc-119(+)] II, xkSi2[Ppie-1::henn-1::gfp::tbb-2 3′UTR cb-unc-119(+)] II*; LGIII: *rde-4(ne301), henn-1(tm4477)*; LGIV: *eri-1(mg366)*, *fem-1(hc17)*, *him-8(e1489)*; LGV: *ergo-1(tm1860)*. The *neIs23[unc-119(+) GFP::ALG-3]* strain was generously provided by Craig Mello (University of Massachusetts, Worcester, MA).

### RNA Sample Preparation

For embryo samples, L1 larvae were grown at 20°C until gravid. Embryos were isolated using sodium hypochlorite solution; an aliquot of embryos was allowed to hatch overnight at room temperature to determine viability. For male samples, synchronized *him-8(e1489)* L1 larvae were grown at 20°C for 72–75 hours. Males were isolated by filtering through 35 µm mesh [71]. For female samples, synchronized *fem-1(hc17)* L1 larvae were plated and grown at 25°C for 52 hours. For time course samples, synchronized wild-type (N2) and *henn-1(tm4477)* L1 larvae were grown at 25°C until gravid; embryos were extracted and harvested for RNA or hatched overnight at room temperature and then grown at 25°C for the specified number of hours before harvest. The *prg-1(tm872)* time course samples were prepared in the same way, except that animals were grown for the first generation at 20°C to evade temperature-sensitive sterility. Samples were processed by either three rounds of freeze/thaw lysis or two rounds of homogenization for 15 sec using the Tissue Master-125 Watt Lab Homogenizer (Omni International) and the RNA was extracted in TriReagent (Ambion) following the vendor's protocol, with the following alterations: RNA was precipitated in isopropanol for one hour at −80°C; RNA was pelleted by centrifugation at 4°C for 30 min at 20,000× g; the pellet was washed three times in 75% ethanol; the pellet was resuspended in water.

### β-elimination Assay for Small RNAs and Northern Blot Analysis

For detection of small RNAs, 10 or 40 µg of total RNA were β-eliminated as described [51]; control samples were processed in parallel without sodium periodate. Northern blot analysis was performed as described [72]. In brief, 5 or 10 µg of β-eliminated total RNA were resolved on 17.5% or 20% denaturing Urea-PAGE gels (SequaGel, National Diagnostics) and transferred to Hybond-NX membrane (Amersham). 21 and 26 nt synthetic RNAs were run as size markers and visualized in tandem with rRNA by ethidium bromide staining. Pre-hybridization/hybridization and washes were performed at 48°C or 50°C. Oligonucleotides corresponding to the antisense sequences of the small RNAs (Table S1) were synthesized and end-labeled with [α-^32^P]-dATP using the miRNA StarFire kit (Integrated DNA Technologies).

### RNAi Sensitivity Assay

To test the response to exogenous RNAi, bacterial clones from the Ahringer RNAi library [73] were diluted with bacteria harboring the empty vector *L4440* to achieve a level of RNAi sensitivity that allowed us to differentiate the RNAi responses in the strains examined. To determine *lir-1* RNAi sensitivity, the *lir-1* RNAi bacterial clone diluted with *L4440* bacterial clone at a 1∶1 or 1∶2 ratio (1/2 or 1/3 strength) was used; >50 L1 larvae were plated per plate and the number of total animals assayed per plate was determined at day two after plating; the percent of animals exhibiting the larval arrest phenotype was determined at 70 hours at 20°C. Sensitivity to RNAi of *dpy-13* and *lin-29* was also assessed using this method, where animals subjected to *dpy-13* RNAi were imaged at 70 hours and those subjected to *lin-29* RNAi were evaluated for the absence of protruding vulva or bursting phenotype. For *pos-1* RNAi, synchronized L1 larvae were singled onto plates with *pos-1* RNAi diluted with empty vector at a 1∶2 ratio (1/3 strength) that had been induced overnight at 25°C. Animals were grown at 20°C for six days and progeny were counted. Sensitivity to RNAi of *pie-1*, *par-1*, and *par-2* was assessed similarly at the indicated dilutions with 4 plates of 4 P_0_ animals per strain. Sensitivity to *glp-1* RNAi was determined at the indicated dilutions by plating 4 plates of >50 L1 larvae per strain per gene and scoring for the absence of oocytes and embryos in both arms of the germline at 70 hours at 20°C. For all RNAi sensitivity assays, data are representative of at least two independent experiments.

### Fertility Assay

To determine brood size, synchronized L1 larvae from gravid adults grown at 20°C or shifted to 25°C for two generations were singled onto plates with OP50 and grown to adulthood at their respective temperatures. Once egg-laying began, animals (N≥13 per strain) were transferred to fresh plates daily until the supply of fertilized eggs was exhausted. Progeny of the singled parents were counted as late larvae/adults. Results are representative of two independent experiments.

### Quantitative RT–PCR

Taqman small RNA probes were synthesized by Applied Biosystems (Table S2) [74]. For each reaction, 50 ng of total RNA were converted into cDNA using Multiscribe Reverse Transcriptase (Applied Biosystems). The resulting cDNAs were analyzed by a Realplex thermocycler (Eppendorf) with TaqMan Universal PCR Master Mix, No AmpErase UNG (Applied Biosystems). We could not identify a small RNA whose levels were consistent across development for use in normalization. Therefore, to preserve the developmental profile of each of the small RNA assessed, back transformation was used to calculate relative small RNA levels from qRT-PCR cycle numbers. As a control for RNA quality, miR-1 Taqman assays were run in parallel for all samples excluding the ERGO-1 RNA immunoprecipitation samples, in which miRNAs are absent. For quantification of mRNA levels, 100 ng of total RNA were converted into cDNA with Multiscribe Reverse Transcriptase (Applied Biosystems) following the vendor's protocol with the following changes: 25 units of RT and 7.6 units of RNAse OUT (Invitrogen) were used per reaction. cDNAs were analyzed using Power Sybr Green PCR Master Mix (Applied Biosystems) (primers, Table S3). Relative mRNA levels were calculated based on the ΔΔ2*C*_t_ method [75] using *eft-2* for normalization. For all qPCR, 40 cycles of amplification were performed; reactions whose signals were not detected were therefore assigned a cycle number of 40. All results presented are the average values of independent calculations from biological triplicates unless indicated. To determine average upregulation of ERGO-1 26G RNA targets in *henn-1* relative to wild-type (Figure 5C), the mean was calculated for all of the ratios generated by dividing each *henn-1* biological replicate by each wild-type biological replicate.

### 3′ RACE

3′ RACE was performed using the 3′ RACE System for Rapid Amplification of cDNA ends (Invitrogen) according to the manufacturer's protocol. *henn-1* gene-specific primer (5′ GCAGTATGTCGCCTCCAAGTAGAT 3′) was used to amplify *henn-1* 3′ ends from cDNA generated from embryo. Product corresponding to only the seven-exon gene model of *henn-1* was observed, consistent with detection of a single protein isoform corresponding to this model on western blot analysis.

### Plasmids and Transgenic Strains

The endogenous *henn-1::gfp* reporter construct *(xkSi1)* was generated by introducing the following fragments into pCFJ151: endogenous promoter of the *henn-1*-containing operon CEOP3488 [76] (3.9 kb PCR fragment immediately upstream of the *C30A5.3* start codon), *henn-1* genomic coding region (1.8 kb PCR fragment with mutated termination codon), *gfp* coding region (0.9 kb fragment with multiple synthetic introns and termination codon), and *henn-1* endogenous 3′UTR (1.1 kb PCR fragment immediately downstream of *henn-1* termination codon). The germline-only *henn-1::gfp* reporter construct (*xkSi2*) was generated as above with the following substitutions: CEOP3488 operon promoter was replaced with the *pie-1* promoter (2.4 kb PCR fragment immediately upstream of *pie-1* start codon) and *henn-1* endogenous 3′UTR was replaced with the *C36E8.4* 3′UTR (0.3 kb PCR fragment downstream of *C36E8.4*). Constructs were cloned into the pCFJ151 vector, confirmed by sequencing, and used to generate single-copy integrated transgenes via the MosSCI technique [52]. Gene fusion products of the expected size were specifically detected by western blot with both anti-HENN-1 and anti-GFP antibodies.

### Generation of Antibodies

Synthetic antigenic peptides were conjugated to KLH and each was used to immunize two rabbits (Proteintech). Antisera were subsequently affinity purified using Affi-Gel 15 gel (Bio-Rad). Antigenic peptide sequences are as follows: N-terminal HENN-1 peptide with N-terminal added cysteine (CTYVEAYEQLEIALLEPLDR), C-terminal ERGO-1 peptide (CEVNKDMNVNEKLEGMTFV).

### Western Blot Analysis

Proteins immobilized on Immobilon-FL transfer membrane (Millipore) were probed with anti-HENN-1 rabbit polyclonal antibody (1∶2000), anti-γ-tubulin rabbit polyclonal antibody (LL-17) (Sigma) (1∶2000), or anti-ERGO-1 rabbit polyclonal antibody (1∶1000). Peroxidase-AffiniPure goat anti-rabbit IgG secondary antibody was used at 1∶10000 (Jackson ImmunoResearch Laboratories) for detection using Pierce ECL Western Blotting Substrate (Thermo Scientific).

### Isolation of ERGO-1–Associated RNAs

Wild-type, *henn-1*, or *eri-1(mg366)* embryos isolated from gravid adults grown at 20°C were frozen in liquid nitrogen and homogenized with a Mixer Mill MM 400 ball mill homogenizer (Retsch) Homogenates were suspended in lysis buffer (50 mM HEPES (pH 7.4), 1 mM EGTA, 1 mM MgCl_2_, 100 mM KCl, 10% glycerol, 0.05% NP-40 treated with a Complete, Mini, EDTA-free Protease Inhibitor Cocktail tablet (Roche Applied Sciences)) and clarified by centrifugation at 12,000× g for 12 minutes at 4°C. Aliquots of homogenate were reserved as crude lysate for western blot to confirm that immunoprecipitations were performed in lysates of equivalent protein concentration (2 mg/mL). For immunoprecipitations, embryo homogenates were incubated at 4°C for one hour with 75 µg anti-ERGO-1 rabbit polyclonal antibody conjugated to Dynabeads Protein A (Invitrogen), after which the beads were washed (500 mM Tris-HCl (pH 7.5), 200 mM KCl, 0.05% NP-40) and associated proteins were eluted with 200 µL glycine. Three quarters of each eluate were precipitated overnight at 4°C in trichloroacetic acid, pelleted, washed with acetone, and resuspended for western blot analysis. The remaining eluate was treated with 2 mg/ml Proteinase K (Roche) and incubated at 37°C for 30 minutes. RNA was isolated from the eluate by incubation with TriReagent and processed as described above. RNA pellets were resuspended in 10 µL water and 5 µL were used for each Taqman RT reaction.

### Immunostaining

Primary antibodies were applied according to the following specifications: anti-GFP mouse monoclonal antibody 3E6 (Invitrogen) was diluted 1∶1500 to detect HENN-1::GFP and 1∶200 to detect ALG-3::GFP; anti-ERGO-1 rabbit polyclonal was diluted 1∶200; and anti-HENN-1 rabbit polyclonal antibody was preabsorbed as described [77] with *henn-1(tm4477)* mutant extract and diluted 1∶200. Alexa Fluor 555 goat anti-rabbit IgG and Alexa Fluor 488 goat anti-mouse IgG (Molecular Probes) secondary antibodies were diluted 1∶500. All antibodies were diluted in 0.5% bovine serum albumin (Sigma). For immunostaining of gonads and embryos, synchronized gravid hermaphrodites or adult males grown at 20°C were dissected on Superfrost Plus positively charged slides (Fisherbrand) with 27 G×1/2 inch BD PrecisionGlide needles (Becton, Dickinson and Company) as described by Chan and Meyer in WormBook [78] Protocol 21 with 1.5% paraformaldehyde (Sigma). Slides were incubated with primary antibodies overnight at 4°C and with secondary antibodies for three hours at room temperature. Slides were mounted with VECTASHIELD Mounting Medium with DAPI (Vector Laboratories). For whole-worm immunostaining, synchronized late L4 larvae grown at 20°C were transferred to subbed slides [77] in M9, fixed for six minutes in 1.5% paraformaldehyde, freeze-cracked, and incubated for 15 minutes in ice cold methanol. After fixation, slides were processed as above. Images were captured on an Olympus BX61 epifluorescence compound microscope with a Hamamatsu ORCA ER camera using Slidebook 4.0.1 digital microscopy software (Intelligent Imaging Innovations) and processed using ImageJ.

## Supporting Information

Figure S1 Alignment of HEN1 Orthologs. A) *C. elegans* HENN-1 bears the conserved HEN1 methyltransferase domain. Protein sequences of HEN1 orthologs from *Caenorhabditis elegans* (NP_741250.1), *Drosophila melanogaster* (NP_610732.1), *Danio rerio* (NP_001017842.1), *Mus musculus* (NP_079999.2), *Homo sapiens* (NP_001096062.1), and *Arabidopsis thaliana* (NP_567616.1) were aligned using T-Coffee [79], [80] with default parameters. The resulting multiple sequence alignment was cropped to show the conserved HEN1 methyltransferase domain (underlined in red) and the C terminus. Significant alignment was not observed for the N terminus. B) Conservation of the HEN1 methyltransferase domain of HENN-1 is comparable to that of other orthologs. Percent identity was calculated using ClustalW (version 2.1; http://www.ebi.ac.uk/Tools/msa/clustalw2/) [81], [82] with default parameters to perform pairwise alignments of the conserved HEN1 methyltransferase domains as defined in Figure S1A.
(TIF)

Figure S2 *C02F5.6* Alleles and Transgenes. A) anti-HENN-1 polyclonal antibody recognizes a single ∼52 kD HENN-1 isoform in wild-type embryo lysate; no protein product is detected in *henn-1(tm4477)* embryo lysate. B) *C02F5.6* (*henn-1*) gene structure showing the encoded N-terminal epitope for generating the anti-HENN-1 rabbit polyclonal antibody, conserved HEN1 domain (pink), and deletion region for the *henn-1(tm4477)* allele (red underline). Aberrant splicing of *henn-1(tm4477)* mRNA is diagrammed below. Activation of a cryptic splice donor site in the *henn-1(tm4477)* mRNA produces a premature termination codon (stop). C) Diagrams of *xkSi1* (endogenous expression) and *xkSi2* (germline-only expression) *henn-1::gfp* transgenes. Transgenes were inserted as single copies on chromosome II via the MosSCI technique [52].
(TIF)

Figure S3 Methylation Status of Additional Small RNAs. A) Additional 21U RNAs show HENN-1-dependent methylation. β-eliminated (βe +) or control treated (βe −) embryo RNA of the indicated genotypes was probed for the specified 21U RNAs. Below, ethidium bromide staining of 5.8S rRNA. *prg-1(tm872)* lacks 21U RNAs and is included as a negative control. B) Additional ERGO-1 class 26G RNAs show HENN-1-dependent methylation in embryo RNA. *eri-1(mg366)* lacks 26G RNAs and is included as a negative control. C) ALG-3/ALG-4 class 26G RNA 26G-S7 shows absence of methylation in *him-8(e1489)* male RNA.
(TIF)

Figure S4 Diverse 21U RNAs Exhibit HENN-1 Dependence in Early Development and Adulthood. A) A panel of additional 21U RNAs exhibit significant defects in accumulation in the *henn-1(tm4477)* mutant. 21U RNA levels were assayed by Taqman qPCR in wild-type and *henn-1(tm4477)* mutant animals at the indicated developmental time points. Standard deviation is shown for biological triplicates. B) 21U RNAs are generally depleted in the *henn-1(tm4477)* mutant relative to wild-type in embryo, early larva, and gravid adult. Abundance in *henn-1(tm4477)* mutant relative to wild-type was calculated for the 21U RNAs shown in A) and Figure 2A and the average was plotted for each time point to illustrate the general effect of loss of *henn-1*. C) 21U RNA Taqman assays specifically detect piRNAs. 21U RNA and miRNA levels were assayed in *prg-1(tm872)* mutant embryo biological duplicates. Fold levels relative to wild-type embryo are plotted. E, embryo.
(TIF)

Figure S5 miRNAs Do Not Exhibit HENN-1 Dependence. A) miRNAs are generally unaffected in the *henn-1(tm4477)* mutant. miRNA levels were assayed by Taqman qPCR in wild-type and *henn-1(tm4477)* mutant animals at the developmental time points assessed in Figure S4. Standard deviation is shown for biological triplicates. B) miRNAs are not generally depleted in the *henn-1(tm4477)* mutant relative to wild-type. Abundance in *henn-1(tm4477)* mutant relative to wild-type was calculated for the miRNAs shown in A) and the average was plotted for each time point to illustrate the general effect of loss of *henn-1*. E, embryo.
(TIF)

Figure S6 HENN-1 Dependence of Substrate-dependent Secondary siRNAs. A) Levels of a Wago-dependent, 21U RNA-dependent 22G RNA targeting *Tc3* are decreased in *henn-1(tm4477)* mutant embryo (P = 0.0064; two-tailed *t*-test). Standard deviation is shown for biological triplicates. B) Levels of a Wago-dependent, ERGO-1 class 26G RNA-dependent 22G RNA targeting *E01G4.7* are decreased in *henn-1(tm4477)* mutant embryo (P = 0.044; two-tailed *t*-test). Standard deviation is shown for biological triplicates.
(TIF)

Figure S7 *henn-1* Contributes to Robust Fertility at Elevated Temperatures. *henn-1(tm4477)* mutant animals exhibit a modest fertility defect at 25°C that is rescued by germline-specific expression of *henn-1::gfp* from transgene *xkSi2*. Progeny per animal cultured at 20°C or shifted to 25°C for three generations is plotted for animals of the indicated genotype. Differences between *henn-1(tm4477)* mutant and wild-type or *xkSi2; henn-1(tm4477)* transgenic rescue strain are statistically significant (*: P = 0.0059; **: P = 0.0130, two-tailed *t*-test). N≥13 animals per strain. Mean and standard deviation are shown.
(TIF)

Figure S8 Many ERGO-1 Class 26G RNAs Exhibit HENN-1 Dependence throughout Development. A) A panel of additional ERGO-1 class 26G RNAs exhibit significant defects in accumulation in the *henn-1(tm4477)* mutant. ERGO-1 class 26G RNA levels were assayed by Taqman qPCR in wild-type and *henn-1(tm4477)* mutant animals at the indicated developmental time points. Standard deviation is shown for biological triplicates. B) ERGO-1 class 26G RNAs are generally depleted in the *henn-1(tm4477)* mutant relative to wild-type throughout development. Abundance in *henn-1(tm4477)* mutant relative to wild-type was calculated for the 26G RNAs shown in A) and Figure 5A and the average was plotted for each time point to illustrate the general effect of loss of *henn-1*. C) ERGO-1 class 26G RNA Taqman assays specifically detect ERI-1-dependent small RNAs. ERGO-1 class 26G RNA and miRNA levels were assayed in *eri-1(mg366)* mutant embryo biological duplicates. Fold levels relative to wild-type embryo are plotted. E, embryo.
(TIF)

Figure S9 ALG-3/ALG-4 Class 26G RNAs Do Not Exhibit HENN-1 Dependence. A) Additional ALG-3/ALG-4 class 26G RNAs do not exhibit significant defects in accumulation in the *henn-1(tm4477)* mutant. ALG-3/ALG-4 class 26G RNA levels were assayed by Taqman qPCR in wild-type and *henn-1(tm4477)* mutant animals at the indicated developmental time points. Standard deviation is shown for biological triplicates. B) ALG-3/ALG-4 class 26G RNAs are generally unchanged in the *henn-1(tm4477)* mutant relative to wild-type during their peak expression. Abundance in *henn-1(tm4477)* mutant relative to wild-type was calculated for the 26G RNAs shown in A) and Figure 5B and the average was plotted for each time point to illustrate the general effect of loss of *henn-1*. C) ALG-3/ALG-4 class 26G RNA Taqman assays specifically detect ERI-1-dependent small RNAs. ALG-3/ALG-4 class 26G RNA and miRNA levels were assayed in *eri-1(mg366); him-8(e1489)* mutant male biological duplicates. Fold levels relative to wild-type male are plotted. D) miRNAs are generally unaffected in the *henn-1(tm4477)* mutant. miRNA levels were assayed by Taqman qPCR in wild-type and *henn-1(tm4477)* mutant animals at the developmental time points assessed in A. Standard deviation is shown for biological triplicates. B) miRNAs are not generally depleted in *henn-1(tm4477)* mutant relative to wild-type animals. Abundance in *henn-1(tm4477)* mutant relative to wild-type was calculated for the miRNAs shown in D) and the average was plotted for each time point to illustrate the general effect of loss of *henn-1*.
(TIF)

Figure S10 The *henn-1(tm4477)* Mutant Does Not Exhibit Significant Upregulation of ERGO-1 Class 26G RNA Target mRNAs. A) ERGO-1 class 26G RNA target mRNAs show only sporadic HENN-1 dependence. Data is summarized in Figure 5C. Levels of eight ERGO-1 class 26G RNA targets were assayed across development of wild-type and *henn-1(tm4477)* mutant animals at 25°C and normalized to mRNA levels of *eft-2*, an abundantly expressed housekeeping gene. Standard deviation is shown for biological triplicates. B) Non-target mRNAs do not show upregulation in the *henn-1(tm4477)* mutant relative to wild-type. Levels of two non-target mRNAs were assayed across development of wild-type and *henn-1(tm4477)* mutant animals at 25°C and normalized to *eft-2*. Standard deviation is shown for biological triplicates. E, embryo.
(TIF)

Figure S11 The *henn-1(tm4477)* Mutant Exhibits a Mild but General Somatic Eri Phenotype. A) *henn-1(tm4477)* mutant animals are weakly somatic Eri to RNAi knockdown of *dpy-13*. Animals of the indicated genotypes were plated as L1 larvae on *dpy-13* feeding RNAi diluted 1∶2 or 1∶5 (1/3 or 1/6 strength) with empty vector and grown for 90 hours at 20°C. *eri-1(mg366)* and *rde-4(ne301)* are included as controls. B) *henn-1(tm4477)* mutant animals are weakly somatic Eri to RNAi knockdown of *lin-29*. Animals of the indicated genotypes were plated as L1 larvae on *lin-29* feeding RNAi diluted 1∶2 (1/3 strength) or 1∶5 (1/6 strength) with empty vector and grown for 70 hours at 20°C. Percent of animals reaching full size without exhibiting protruding vulva or bursting is plotted. N = 4 plates of >50 animals per strain. Mean and standard deviation are shown.
(TIF)

Figure S12 The *henn-1(tm4477)* Mutant Exhibits a General Germline Rde Phenotype. A) *henn-1(tm4477)* mutant animals are Rde to RNAi knockdown of germline genes. Animals of the indicated genotypes were plated as L1 larvae on *par-1*, *par-2*, or *pie-1* feeding RNAi diluted to the indicated strengths with empty vector and grown for 6 days at 20°C. Brood size averaged to the number of P_0_ L1s per plate is plotted. N = 4 plates of 4 P_0_ animals per strain. Mean and standard deviation are shown. *: P = 0.0234; **: P = 0.0028; ***: P = 0.0151; ****: P = 0.0098, two-tailed *t*-test. B) *henn-1(tm4477)* mutant animals are weakly Rde to RNAi knockdown of germline development gene *glp-1*. Animals of the indicated genotypes were plated as L1 larvae on *glp-1* feeding RNAi and grown for 70 hours at 20°C. Percent of animals failing to develop both arms of the germline is plotted. *rde-4(ne301)* is included as a control. N = 4 plates of >50 animals per strain. Mean and standard deviation are shown. ^†^: P = 0.0424, two-tailed *t*-test.
(TIF)

Figure S13 HENN-1 is Broadly Expressed in the Germline and Soma. A) *henn-1* mRNA is highly expressed throughout development. Non-normalized *henn-1* mRNA levels are plotted relative to *eft-2* mRNA levels. The expression profile of *henn-1* is largely unaffected by normalization to *eft-2* (as shown in Figure 7A). B) *henn-1* is expressed in germline and soma. Levels of *henn-1* mRNA were assayed in wild-type and *glp-4(bn2)* mutant animals grown for 56 hours at 25°C. C) HENN-1::GFP is broadly expressed in both germline and somatic tissues. HENN-1::GFP was detected in *xkSi1; henn-1(tm4477)* L4 larva but not wild-type control larva using anti-GFP mouse monoclonal antibody. D) ERGO-1 and HENN-1::GFP are generally abundant in early embryo; specificity of anti-ERGO-1 antibody in embryo is shown on right. E) ERGO-1 shows cytoplasmic enrichment in the hermaphrodite proximal germline. Extruded gonads of *xkSi1; henn-1(tm4477)* adult hermaphrodite were stained with anti-GFP and anti-HENN-1 antibodies. Staining of *ergo-1(tm1860)* mutant demonstrates specificity of anti-ERGO-1 antibody (right). F) GFP::ALG-3 expression overlaps with that of HENN-1 (inset: residual bodies). Extruded gonads of *gfp::alg-3* transgenic adult males were stained with anti-GFP and anti-HENN-1 antibodies. E, embryo.
(TIF)

Figure S14 Comparison of *C. elegans* Argonautes. A) Phylogram of human, fly, and worm Argonautes shows divergence of CeERGO-1, CeRDE-1, and DmAgo2 relative to other members of their clades. Multiple sequence alignment of the longest annotated RefSeq protein sequences was performed using ClustalW with default parameters and visualized using Phylodendron (version 0.8d; http://www.es.embnet.org/Doc/phylodendron/). Scale, 0.1∶ 0.1 substitutions per site. B) Only Piwi clade Argonautes bear the characteristic PAZ domain insertion. Multiple sequence alignment of select Argonautes was performed using ClustalW with default parameters and cropped to show the context of the PAZ domain insertion between strands β6 and β7 as annotated by Tian et al. [69]. For each Argonaute, methylation status (MET) of associated small RNAs is indicated at right (Yes, methylated; No, not methylated). Sources: HsAGO1, [68], [69] and by analogy to mouse [31]; DmAgo1, [19]; CeALG-1, [19]; CeALG-3, this study; HsPIWIL1, [69] and by analogy to mouse [30], [31]; DmPiwi, [31], [32]; CePRG-1, [27] and this study, DmAgo2, [9], [22]; CeERGO-1, [27], [42] and this study.
(TIF)

Table S1 Oligonucleotides for Northern Blot Analysis. Oligonucleotides corresponding to the antisense sequences of small RNAs were synthesized by Integrated DNA Technologies and used for small RNA detection by northern blot.
(DOC)

Table S2 Small RNA Sequences for Taqman Probe Design. Sequences of the indicated small RNAs were submitted to Applied Biosystems for Taqman small RNA probe design and synthesis.
(DOC)

Table S3 Primers for RT-qPCR. RT-qPCR primers for detection of the indicated gene targets were synthesized by Integrated DNA Technologies.
(DOC)
