# Supplementary figures and images for: The Caenorhabditis elegans HEN1 Ortholog, HENN-1, Methylates and Stabilizes Select Subclasses of Germline Small RNAs
Source: PLoS Genet. 2012 Apr 19;8(4):e1002617. doi: 10.1371/journal.pgen.1002617 (PMC3330095; doi:10.1371/journal.pgen.1002617)

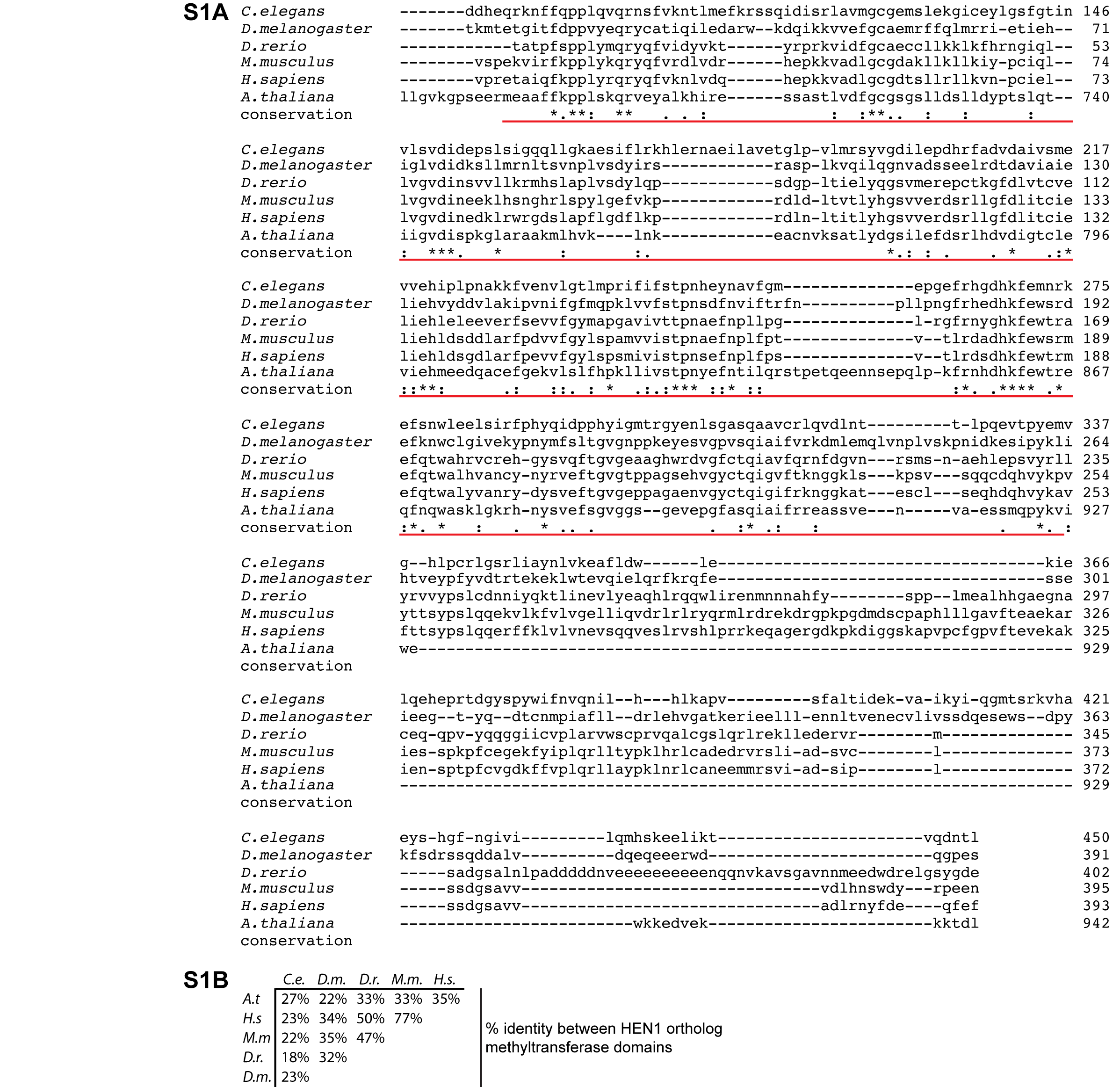

Supplement: Figure S1 — Alignment of HEN1 Orthologs. A) C. elegans HENN-1 bears the conserved HEN1 methyltransferase domain. Protein sequences of HEN1 orthologs from Caenorhabditis elegans (NP_741250.1), Drosophila melanogaster (NP_610732.1), Danio rerio (NP_001017842.1), Mus musculus (NP_079999.2), Homo sapiens (NP_001096062.1), and Arabidopsis thaliana (NP_567616.1) were aligned using T-Coffee [79], [80] with default parameters. The resulting multiple sequence alignment was cropped to show the conserved HEN1 methyltransferase domain (underlined in red) and the C terminus. Significant alignment was not observed for the N terminus. B) Conservation of the HEN1 methyltransferase domain of HENN-1 is comparable to that of other orthologs. Percent identity was calculated using ClustalW (version 2.1; http://www.ebi.ac.uk/Tools/msa/clustalw2/) [81], [82] with default parameters to perform pairwise alignments of the conserved HEN1 methyltransferase domains as defined in Figure S1A. (TIF) [file pgen.1002617.s001.tif]

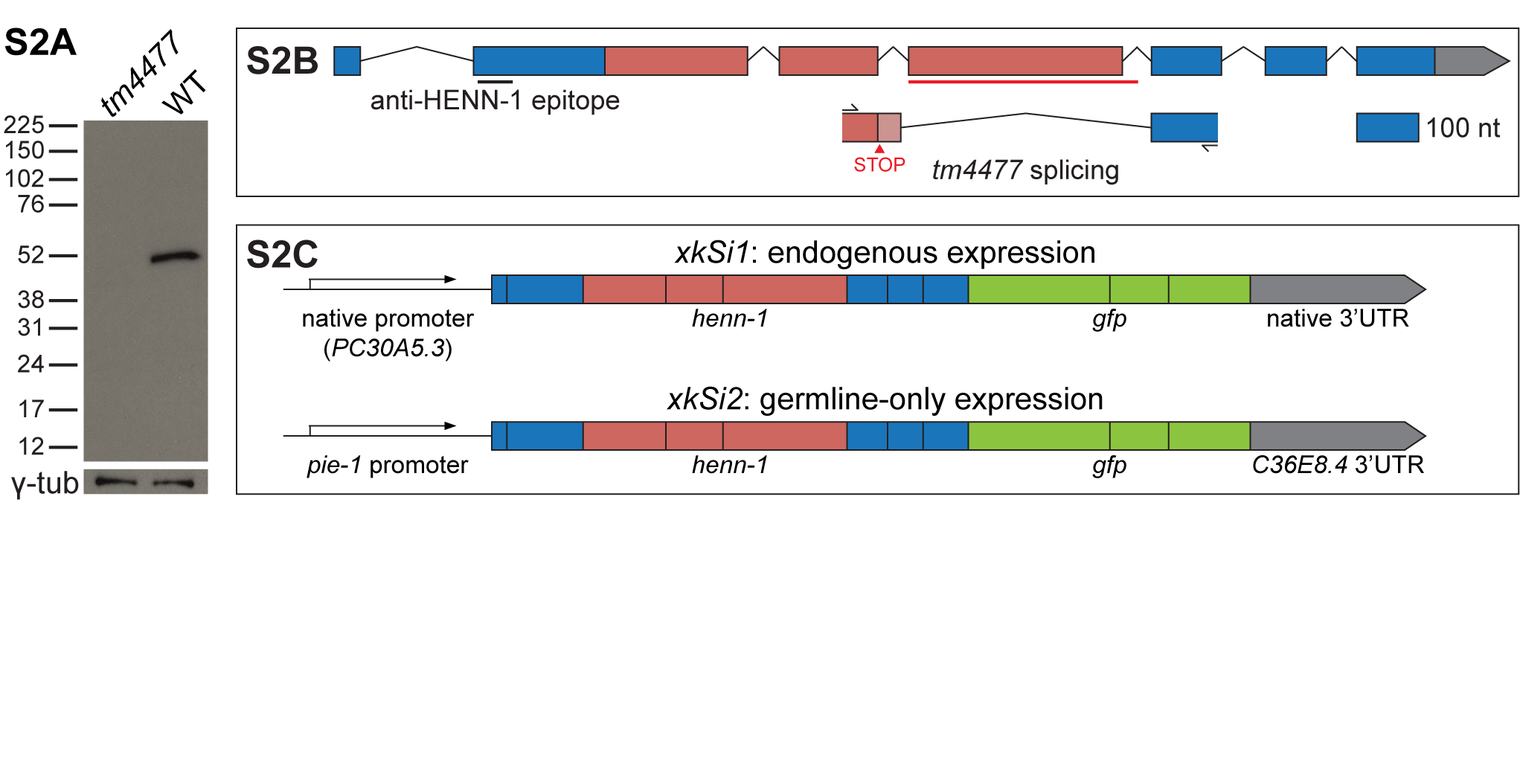

Supplement: Figure S2 — C02F5.6 Alleles and Transgenes. A) anti-HENN-1 polyclonal antibody recognizes a single ∼52 kD HENN-1 isoform in wild-type embryo lysate; no protein product is detected in henn-1(tm4477) embryo lysate. B) C02F5.6 (henn-1) gene structure showing the encoded N-terminal epitope for generating the anti-HENN-1 rabbit polyclonal antibody, conserved HEN1 domain (pink), and deletion region for the henn-1(tm4477) allele (red underline). Aberrant splicing of henn-1(tm4477) mRNA is diagrammed below. Activation of a cryptic splice donor site in the henn-1(tm4477) mRNA produces a premature termination codon (stop). C) Diagrams of xkSi1 (endogenous expression) and xkSi2 (germline-only expression) henn-1::gfp transgenes. Transgenes were inserted as single copies on chromosome II via the MosSCI technique [52]. (TIF) [file pgen.1002617.s002.tif]

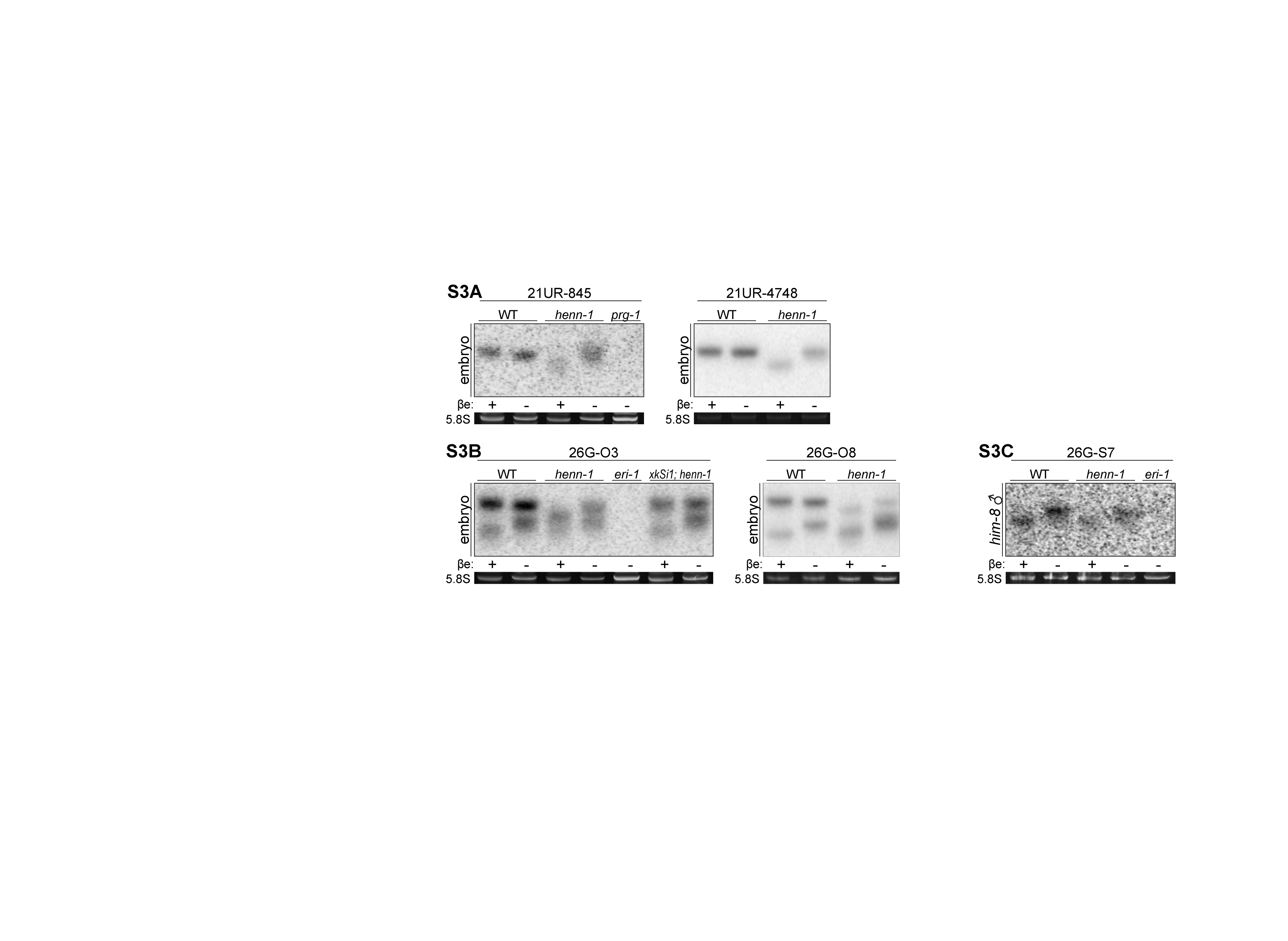

Supplement: Figure S3 — Methylation Status of Additional Small RNAs. A) Additional 21U RNAs show HENN-1-dependent methylation. β-eliminated (βe +) or control treated (βe −) embryo RNA of the indicated genotypes was probed for the specified 21U RNAs. Below, ethidium bromide staining of 5.8S rRNA. prg-1(tm872) lacks 21U RNAs and is included as a negative control. B) Additional ERGO-1 class 26G RNAs show HENN-1-dependent methylation in embryo RNA. eri-1(mg366) lacks 26G RNAs and is included as a negative control. C) ALG-3/ALG-4 class 26G RNA 26G-S7 shows absence of methylation in him-8(e1489) male RNA. (TIF) [file pgen.1002617.s003.tif]

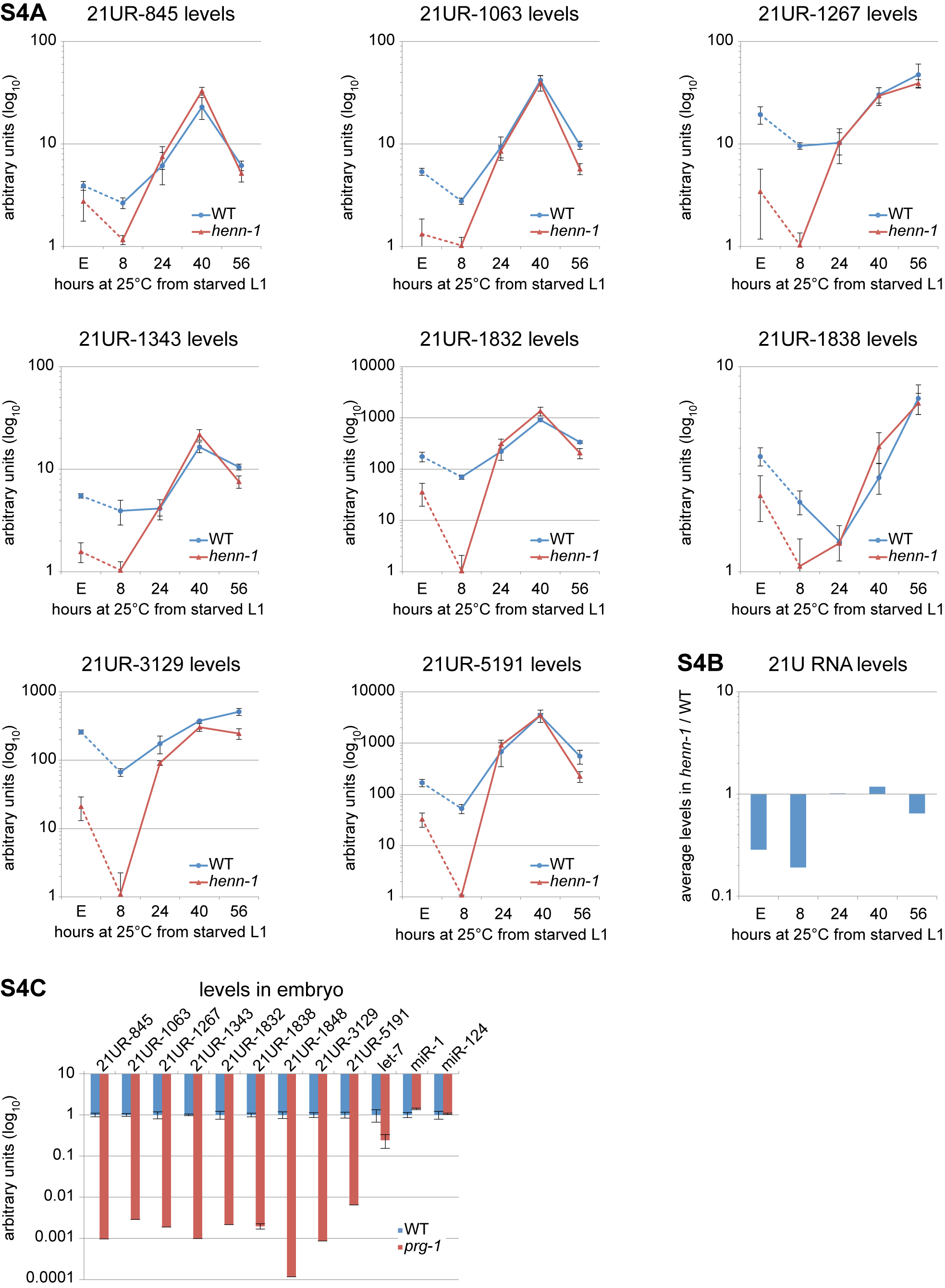

Supplement: Figure S4 — Diverse 21U RNAs Exhibit HENN-1 Dependence in Early Development and Adulthood. A) A panel of additional 21U RNAs exhibit significant defects in accumulation in the henn-1(tm4477) mutant. 21U RNA levels were assayed by Taqman qPCR in wild-type and henn-1(tm4477) mutant animals at the indicated developmental time points. Standard deviation is shown for biological triplicates. B) 21U RNAs are generally depleted in the henn-1(tm4477) mutant relative to wild-type in embryo, early larva, and gravid adult. Abundance in henn-1(tm4477) mutant relative to wild-type was calculated for the 21U RNAs shown in A) and Figure 2A and the average was plotted for each time point to illustrate the general effect of loss of henn-1. C) 21U RNA Taqman assays specifically detect piRNAs. 21U RNA and miRNA levels were assayed in prg-1(tm872) mutant embryo biological duplicates. Fold levels relative to wild-type embryo are plotted. E, embryo. (TIF) [file pgen.1002617.s004.tif]

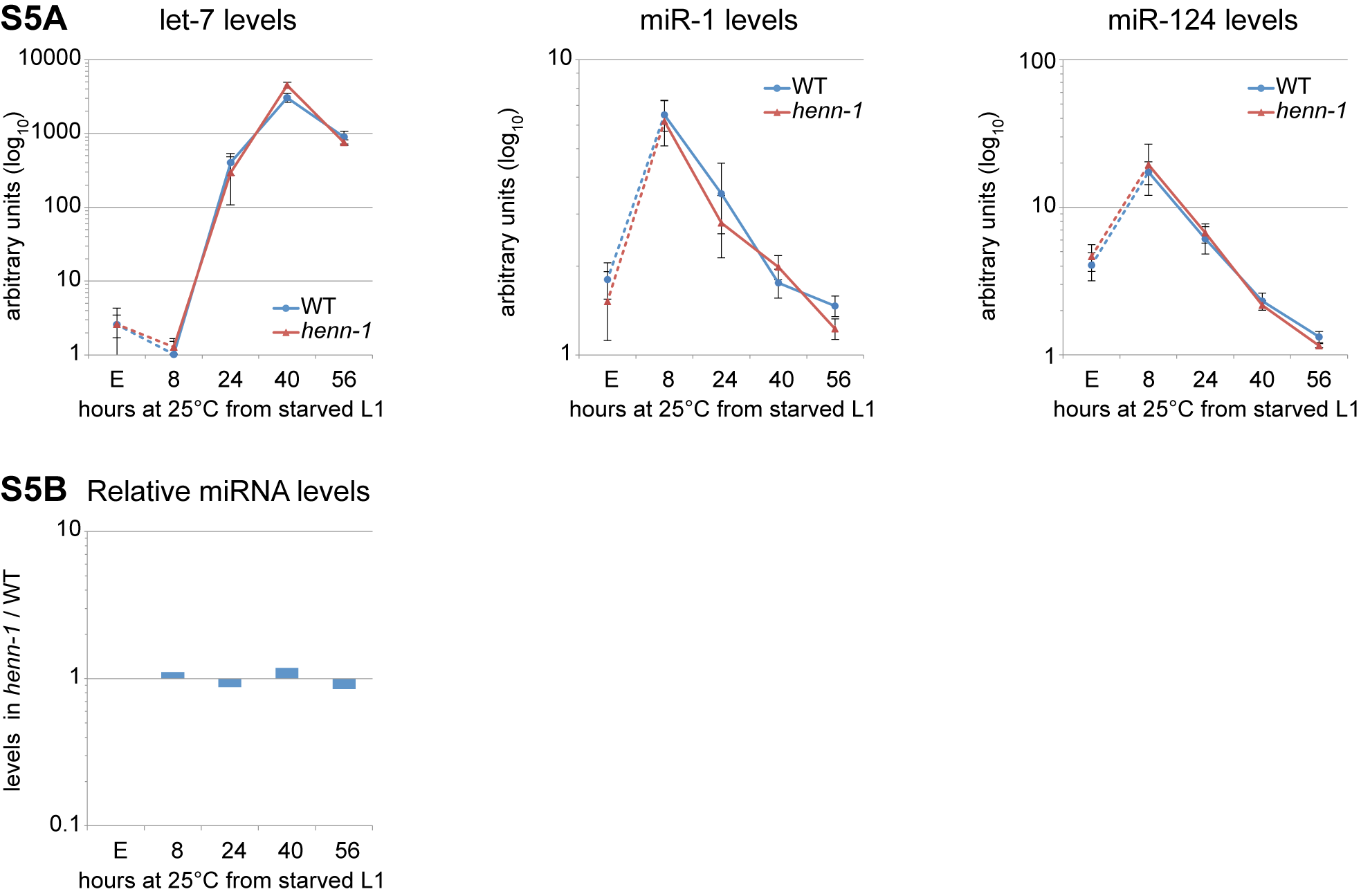

Supplement: Figure S5 — miRNAs Do Not Exhibit HENN-1 Dependence. A) miRNAs are generally unaffected in the henn-1(tm4477) mutant. miRNA levels were assayed by Taqman qPCR in wild-type and henn-1(tm4477) mutant animals at the developmental time points assessed in Figure S4. Standard deviation is shown for biological triplicates. B) miRNAs are not generally depleted in the henn-1(tm4477) mutant relative to wild-type. Abundance in henn-1(tm4477) mutant relative to wild-type was calculated for the miRNAs shown in A) and the average was plotted for each time point to illustrate the general effect of loss of henn-1. E, embryo. (TIF) [file pgen.1002617.s005.tif]

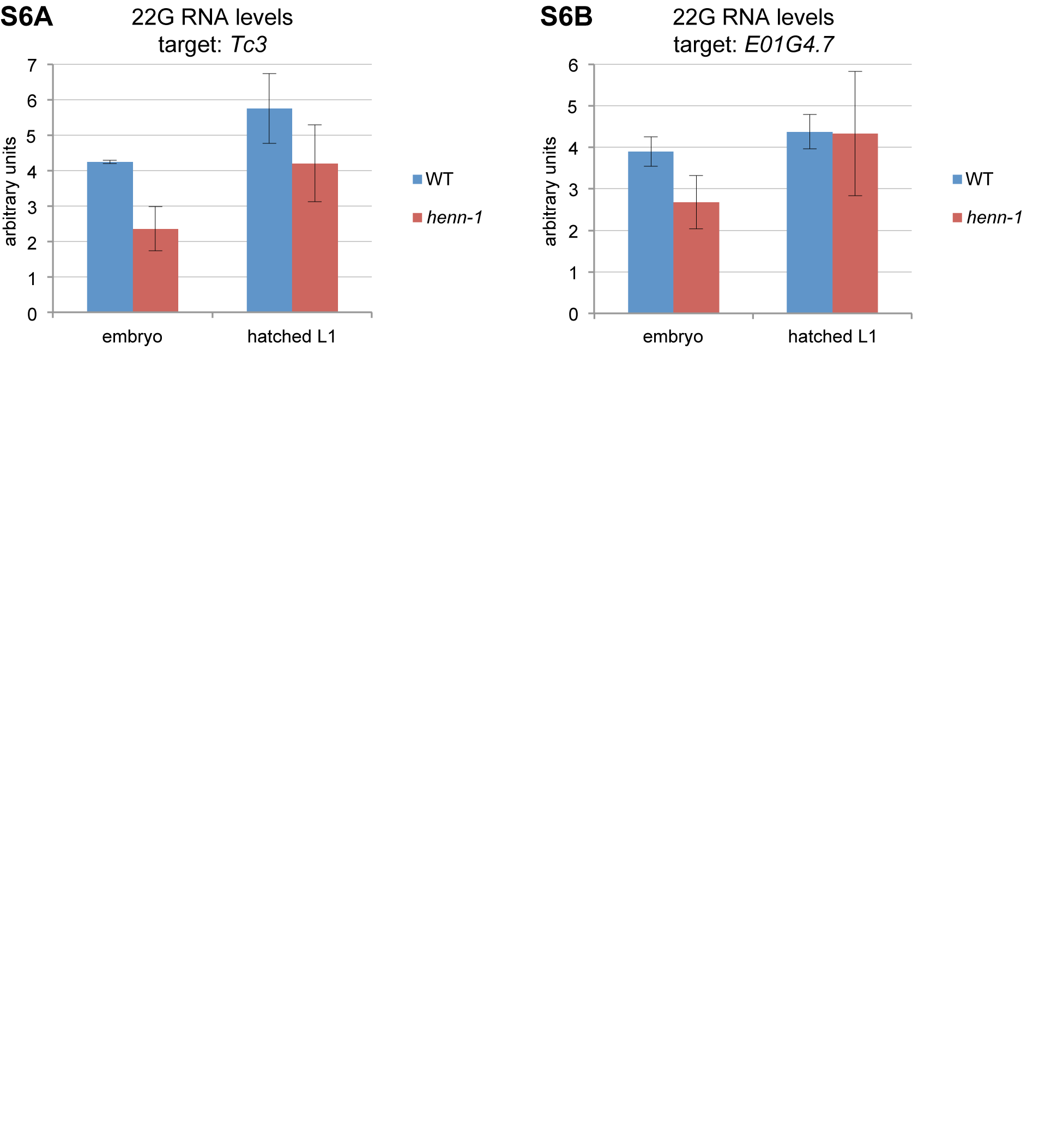

Supplement: Figure S6 — HENN-1 Dependence of Substrate-dependent Secondary siRNAs. A) Levels of a Wago-dependent, 21U RNA-dependent 22G RNA targeting Tc3 are decreased in henn-1(tm4477) mutant embryo (P = 0.0064; two-tailed t-test). Standard deviation is shown for biological triplicates. B) Levels of a Wago-dependent, ERGO-1 class 26G RNA-dependent 22G RNA targeting E01G4.7 are decreased in henn-1(tm4477) mutant embryo (P = 0.044; two-tailed t-test). Standard deviation is shown for biological triplicates. (TIF) [file pgen.1002617.s006.tif]

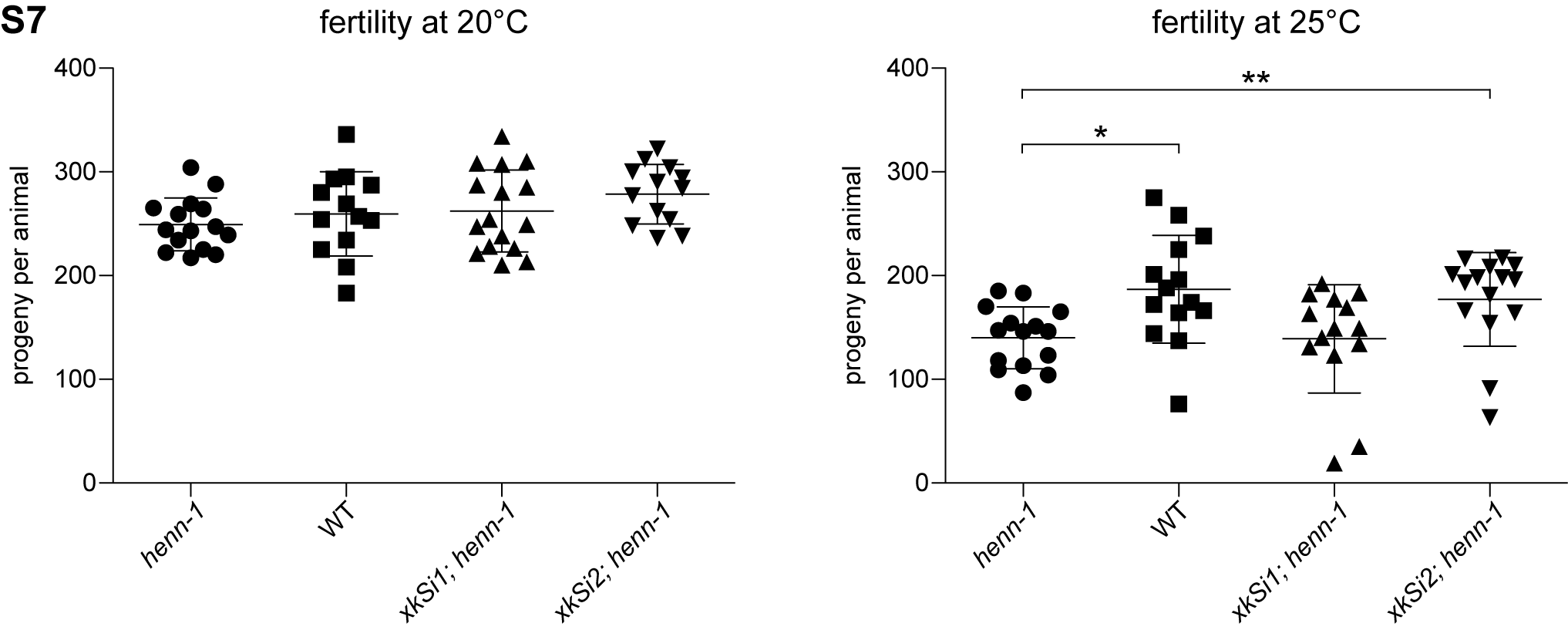

Supplement: Figure S7 — henn-1 Contributes to Robust Fertility at Elevated Temperatures. henn-1(tm4477) mutant animals exhibit a modest fertility defect at 25°C that is rescued by germline-specific expression of henn-1::gfp from transgene xkSi2. Progeny per animal cultured at 20°C or shifted to 25°C for three generations is plotted for animals of the indicated genotype. Differences between henn-1(tm4477) mutant and wild-type or xkSi2; henn-1(tm4477) transgenic rescue strain are statistically significant (*: P = 0.0059; **: P = 0.0130, two-tailed t-test). N≥13 animals per strain. Mean and standard deviation are shown. (TIF) [file pgen.1002617.s007.tif]

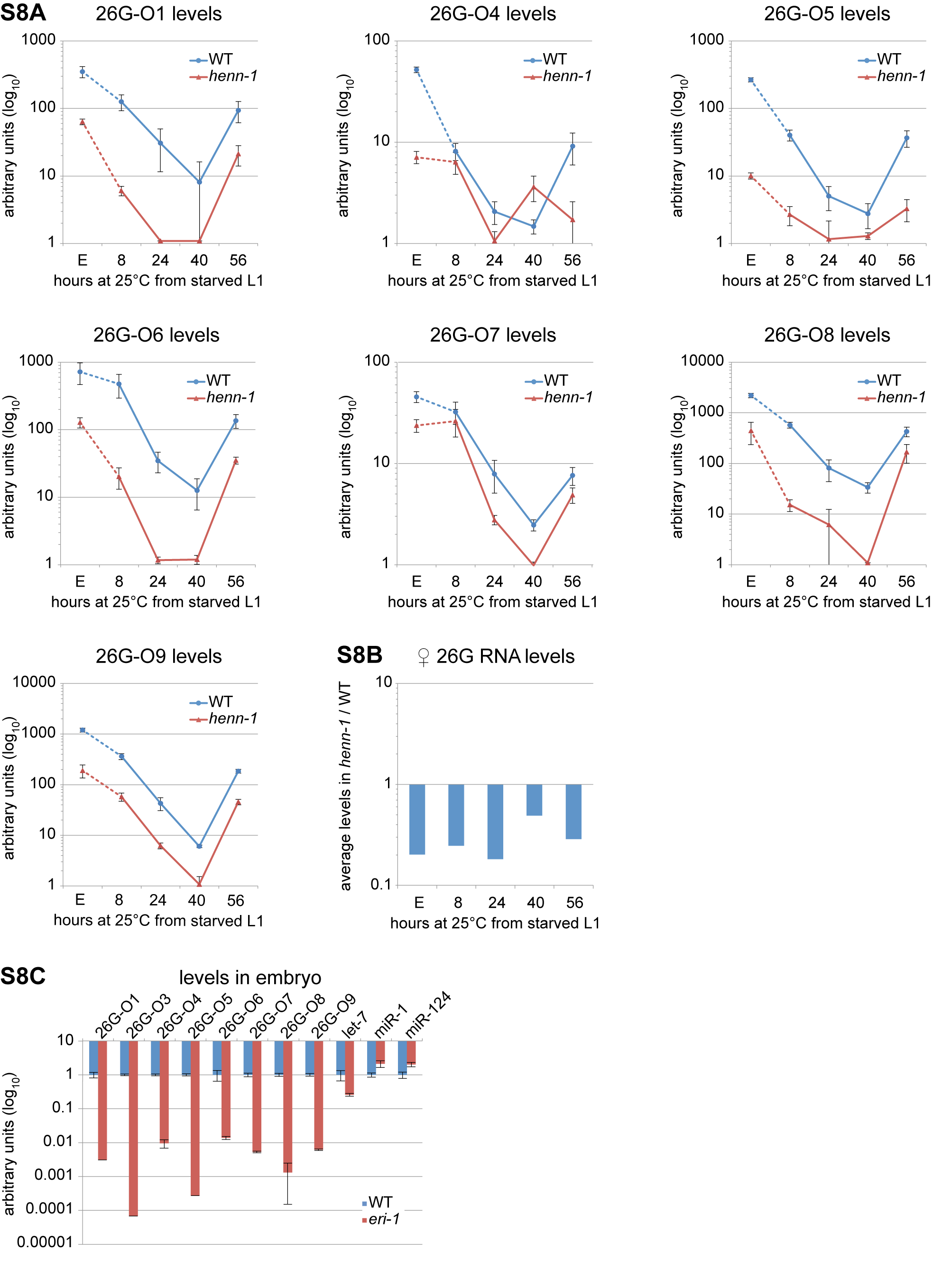

Supplement: Figure S8 — Many ERGO-1 Class 26G RNAs Exhibit HENN-1 Dependence throughout Development. A) A panel of additional ERGO-1 class 26G RNAs exhibit significant defects in accumulation in the henn-1(tm4477) mutant. ERGO-1 class 26G RNA levels were assayed by Taqman qPCR in wild-type and henn-1(tm4477) mutant animals at the indicated developmental time points. Standard deviation is shown for biological triplicates. B) ERGO-1 class 26G RNAs are generally depleted in the henn-1(tm4477) mutant relative to wild-type throughout development. Abundance in henn-1(tm4477) mutant relative to wild-type was calculated for the 26G RNAs shown in A) and Figure 5A and the average was plotted for each time point to illustrate the general effect of loss of henn-1. C) ERGO-1 class 26G RNA Taqman assays specifically detect ERI-1-dependent small RNAs. ERGO-1 class 26G RNA and miRNA levels were assayed in eri-1(mg366) mutant embryo biological duplicates. Fold levels relative to wild-type embryo are plotted. E, embryo. (TIF) [file pgen.1002617.s008.tif]

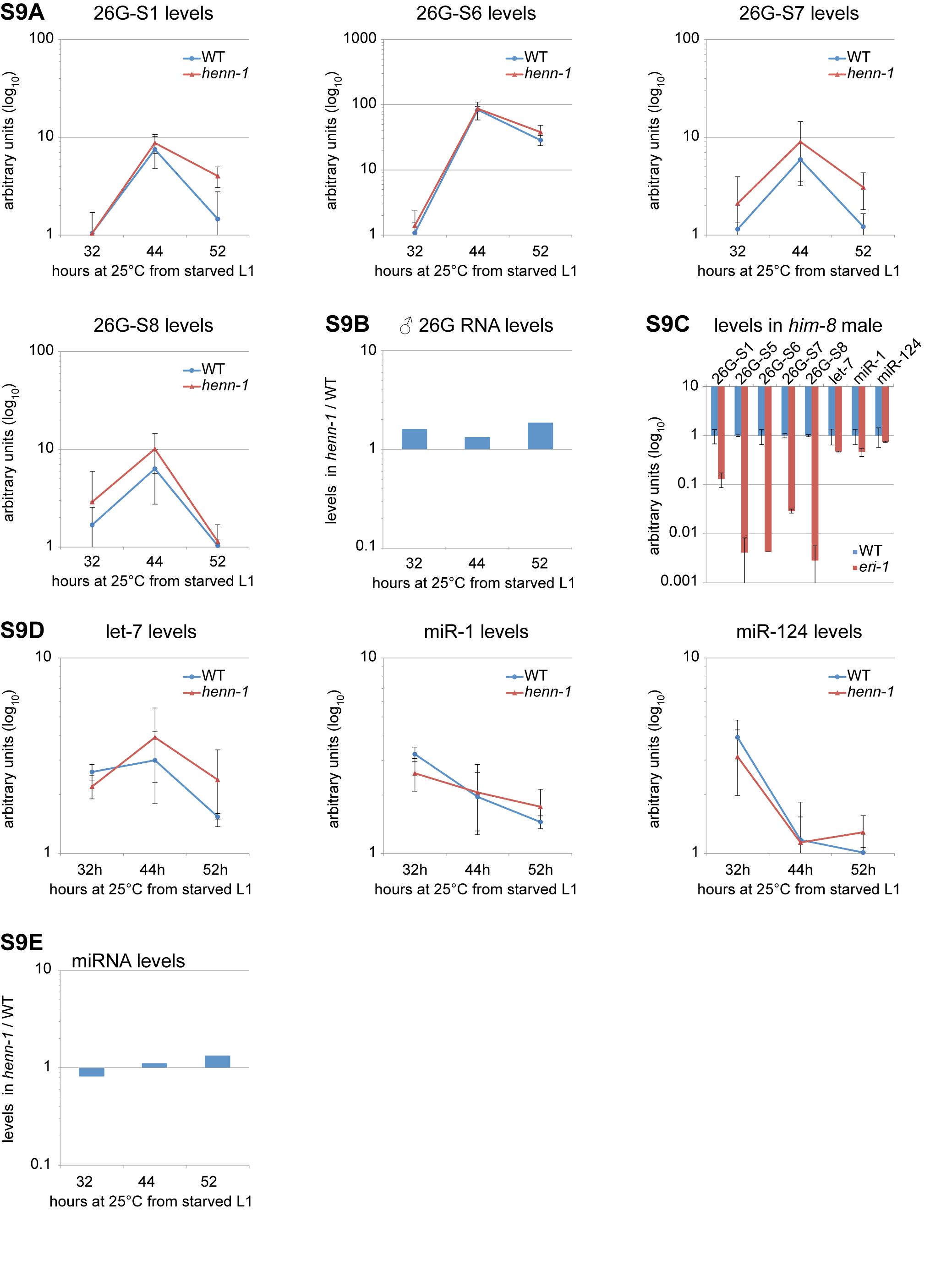

Supplement: Figure S9 — ALG-3/ALG-4 Class 26G RNAs Do Not Exhibit HENN-1 Dependence. A) Additional ALG-3/ALG-4 class 26G RNAs do not exhibit significant defects in accumulation in the henn-1(tm4477) mutant. ALG-3/ALG-4 class 26G RNA levels were assayed by Taqman qPCR in wild-type and henn-1(tm4477) mutant animals at the indicated developmental time points. Standard deviation is shown for biological triplicates. B) ALG-3/ALG-4 class 26G RNAs are generally unchanged in the henn-1(tm4477) mutant relative to wild-type during their peak expression. Abundance in henn-1(tm4477) mutant relative to wild-type was calculated for the 26G RNAs shown in A) and Figure 5B and the average was plotted for each time point to illustrate the general effect of loss of henn-1. C) ALG-3/ALG-4 class 26G RNA Taqman assays specifically detect ERI-1-dependent small RNAs. ALG-3/ALG-4 class 26G RNA and miRNA levels were assayed in eri-1(mg366); him-8(e1489) mutant male biological duplicates. Fold levels relative to wild-type male are plotted. D) miRNAs are generally unaffected in the henn-1(tm4477) mutant. miRNA levels were assayed by Taqman qPCR in wild-type and henn-1(tm4477) mutant animals at the developmental time points assessed in A. Standard deviation is shown for biological triplicates. B) miRNAs are not generally depleted in henn-1(tm4477) mutant relative to wild-type animals. Abundance in henn-1(tm4477) mutant relative to wild-type was calculated for the miRNAs shown in D) and the average was plotted for each time point to illustrate the general effect of loss of henn-1. (TIF) [file pgen.1002617.s009.tif]

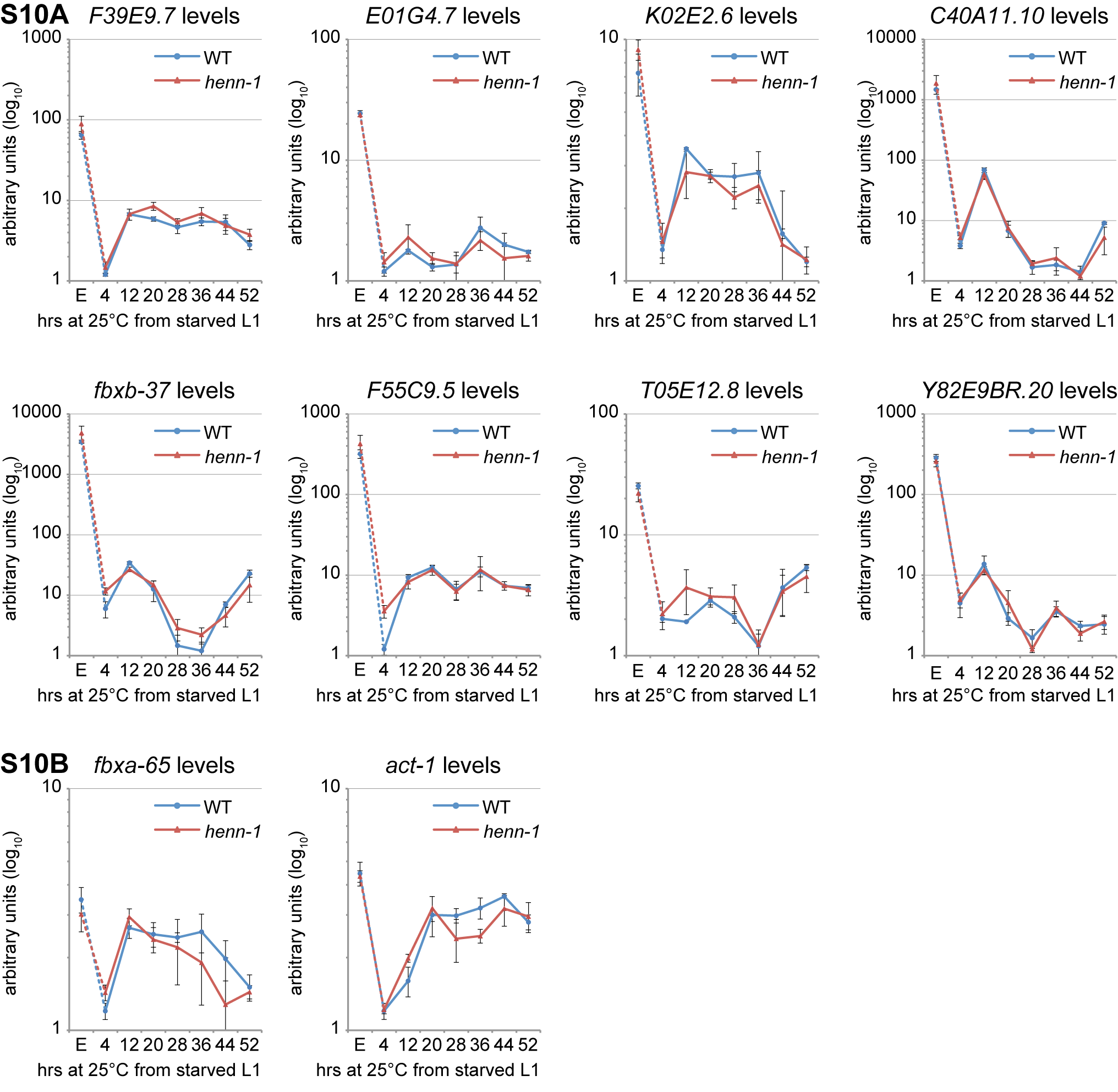

Supplement: Figure S10 — The henn-1(tm4477) Mutant Does Not Exhibit Significant Upregulation of ERGO-1 Class 26G RNA Target mRNAs. A) ERGO-1 class 26G RNA target mRNAs show only sporadic HENN-1 dependence. Data is summarized in Figure 5C. Levels of eight ERGO-1 class 26G RNA targets were assayed across development of wild-type and henn-1(tm4477) mutant animals at 25°C and normalized to mRNA levels of eft-2, an abundantly expressed housekeeping gene. Standard deviation is shown for biological triplicates. B) Non-target mRNAs do not show upregulation in the henn-1(tm4477) mutant relative to wild-type. Levels of two non-target mRNAs were assayed across development of wild-type and henn-1(tm4477) mutant animals at 25°C and normalized to eft-2. Standard deviation is shown for biological triplicates. E, embryo. (TIF) [file pgen.1002617.s010.tif]

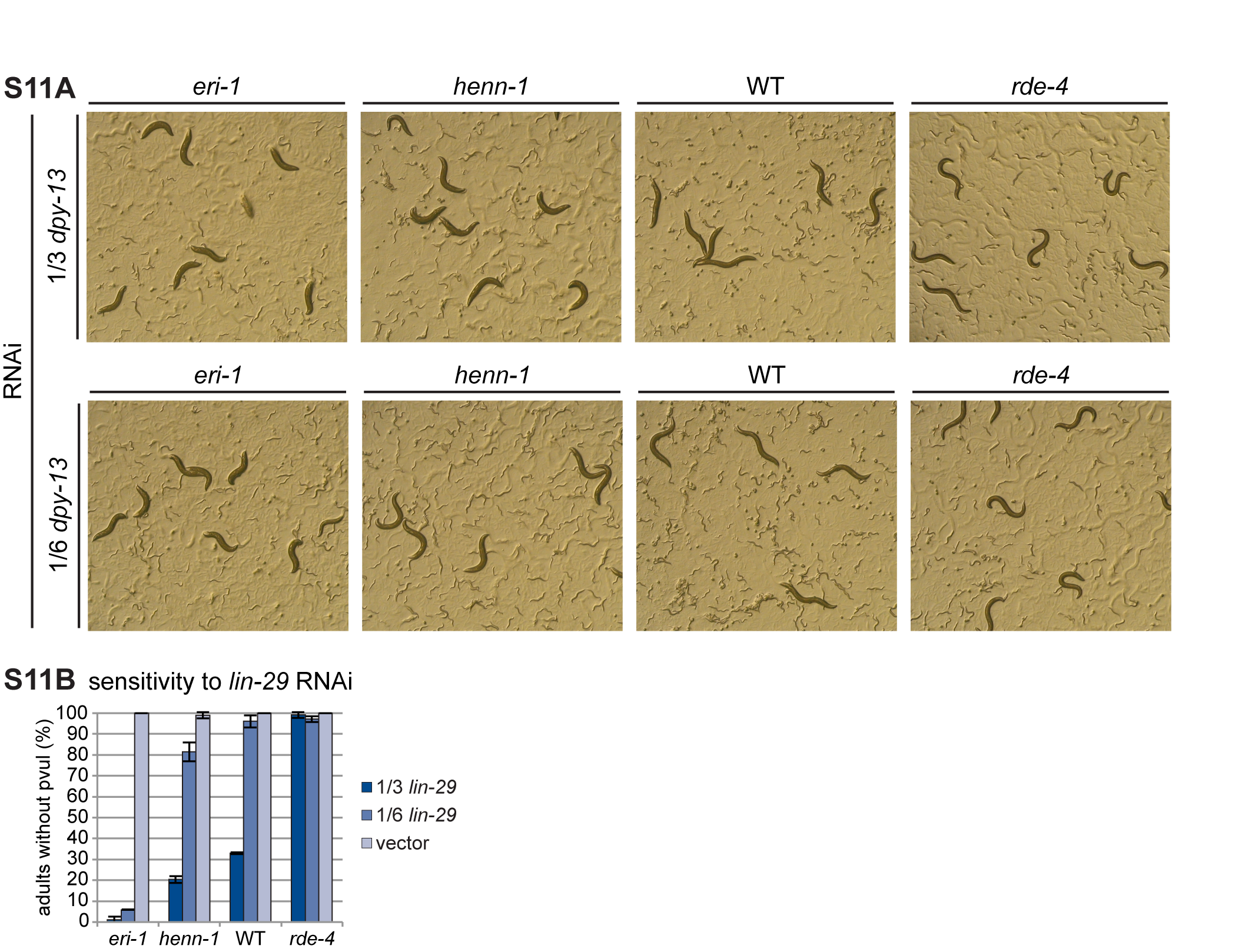

Supplement: Figure S11 — The henn-1(tm4477) Mutant Exhibits a Mild but General Somatic Eri Phenotype. A) henn-1(tm4477) mutant animals are weakly somatic Eri to RNAi knockdown of dpy-13. Animals of the indicated genotypes were plated as L1 larvae on dpy-13 feeding RNAi diluted 1∶2 or 1∶5 (1/3 or 1/6 strength) with empty vector and grown for 90 hours at 20°C. eri-1(mg366) and rde-4(ne301) are included as controls. B) henn-1(tm4477) mutant animals are weakly somatic Eri to RNAi knockdown of lin-29. Animals of the indicated genotypes were plated as L1 larvae on lin-29 feeding RNAi diluted 1∶2 (1/3 strength) or 1∶5 (1/6 strength) with empty vector and grown for 70 hours at 20°C. Percent of animals reaching full size without exhibiting protruding vulva or bursting is plotted. N = 4 plates of >50 animals per strain. Mean and standard deviation are shown. (TIF) [file pgen.1002617.s011.tif]

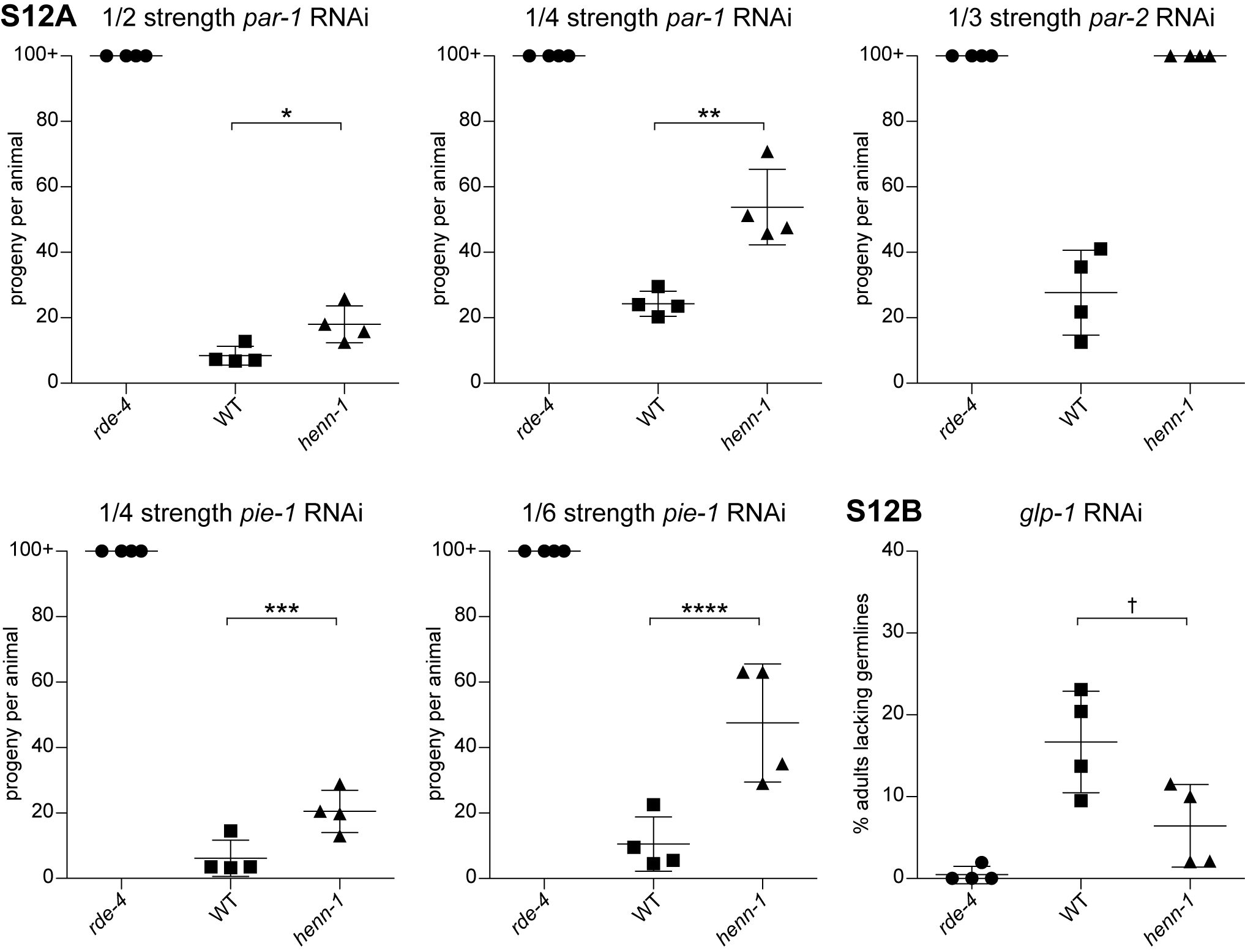

Supplement: Figure S12 — The henn-1(tm4477) Mutant Exhibits a General Germline Rde Phenotype. A) henn-1(tm4477) mutant animals are Rde to RNAi knockdown of germline genes. Animals of the indicated genotypes were plated as L1 larvae on par-1, par-2, or pie-1 feeding RNAi diluted to the indicated strengths with empty vector and grown for 6 days at 20°C. Brood size averaged to the number of P0 L1s per plate is plotted. N = 4 plates of 4 P0 animals per strain. Mean and standard deviation are shown. *: P = 0.0234; **: P = 0.0028; ***: P = 0.0151; ****: P = 0.0098, two-tailed t-test. B) henn-1(tm4477) mutant animals are weakly Rde to RNAi knockdown of germline development gene glp-1. Animals of the indicated genotypes were plated as L1 larvae on glp-1 feeding RNAi and grown for 70 hours at 20°C. Percent of animals failing to develop both arms of the germline is plotted. rde-4(ne301) is included as a control. N = 4 plates of >50 animals per strain. Mean and standard deviation are shown. †: P = 0.0424, two-tailed t-test. (TIF) [file pgen.1002617.s012.tif]

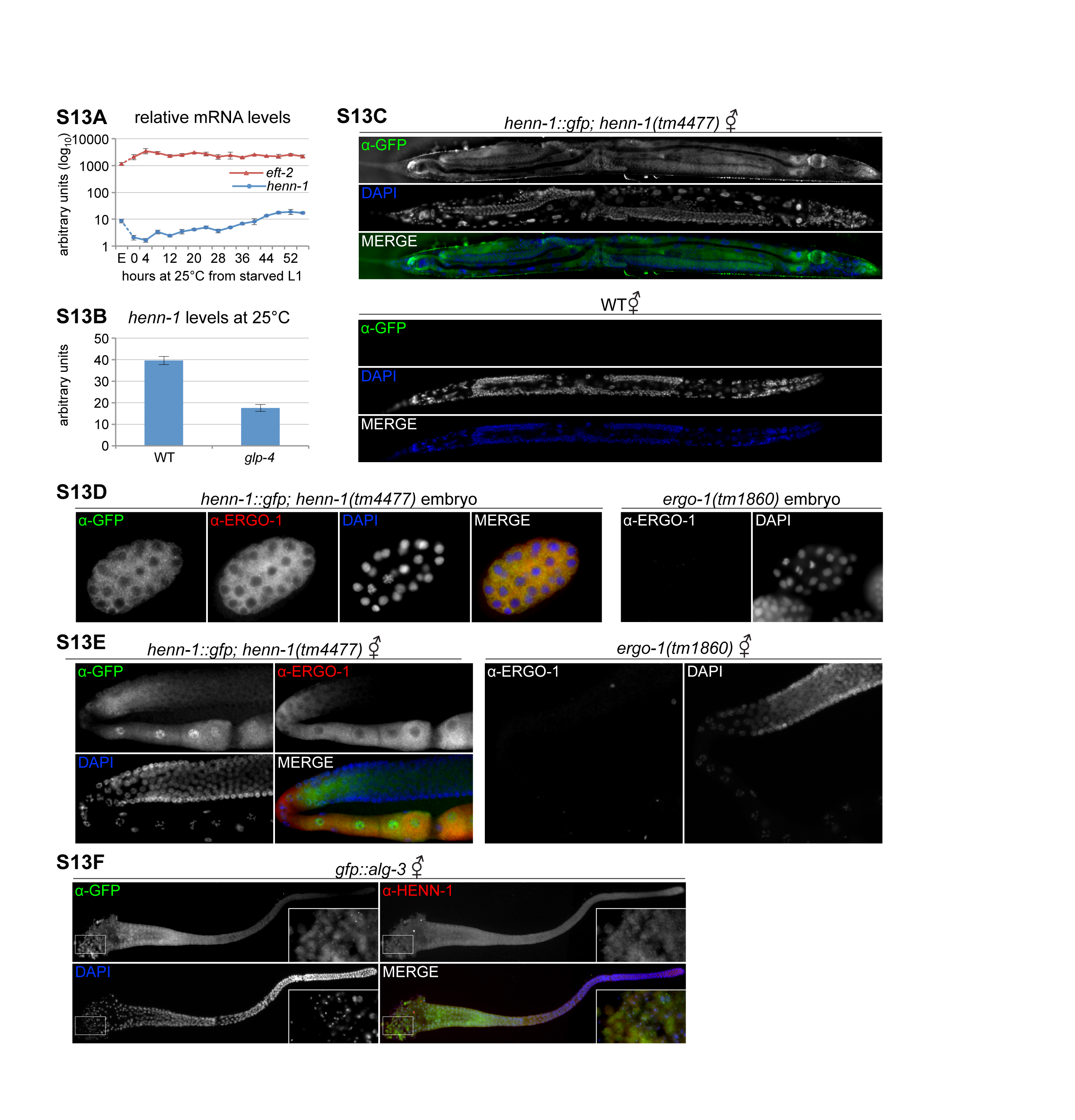

Supplement: Figure S13 — HENN-1 is Broadly Expressed in the Germline and Soma. A) henn-1 mRNA is highly expressed throughout development. Non-normalized henn-1 mRNA levels are plotted relative to eft-2 mRNA levels. The expression profile of henn-1 is largely unaffected by normalization to eft-2 (as shown in Figure 7A). B) henn-1 is expressed in germline and soma. Levels of henn-1 mRNA were assayed in wild-type and glp-4(bn2) mutant animals grown for 56 hours at 25°C. C) HENN-1::GFP is broadly expressed in both germline and somatic tissues. HENN-1::GFP was detected in xkSi1; henn-1(tm4477) L4 larva but not wild-type control larva using anti-GFP mouse monoclonal antibody. D) ERGO-1 and HENN-1::GFP are generally abundant in early embryo; specificity of anti-ERGO-1 antibody in embryo is shown on right. E) ERGO-1 shows cytoplasmic enrichment in the hermaphrodite proximal germline. Extruded gonads of xkSi1; henn-1(tm4477) adult hermaphrodite were stained with anti-GFP and anti-HENN-1 antibodies. Staining of ergo-1(tm1860) mutant demonstrates specificity of anti-ERGO-1 antibody (right). F) GFP::ALG-3 expression overlaps with that of HENN-1 (inset: residual bodies). Extruded gonads of gfp::alg-3 transgenic adult males were stained with anti-GFP and anti-HENN-1 antibodies. E, embryo. (TIF) [file pgen.1002617.s013.tif]

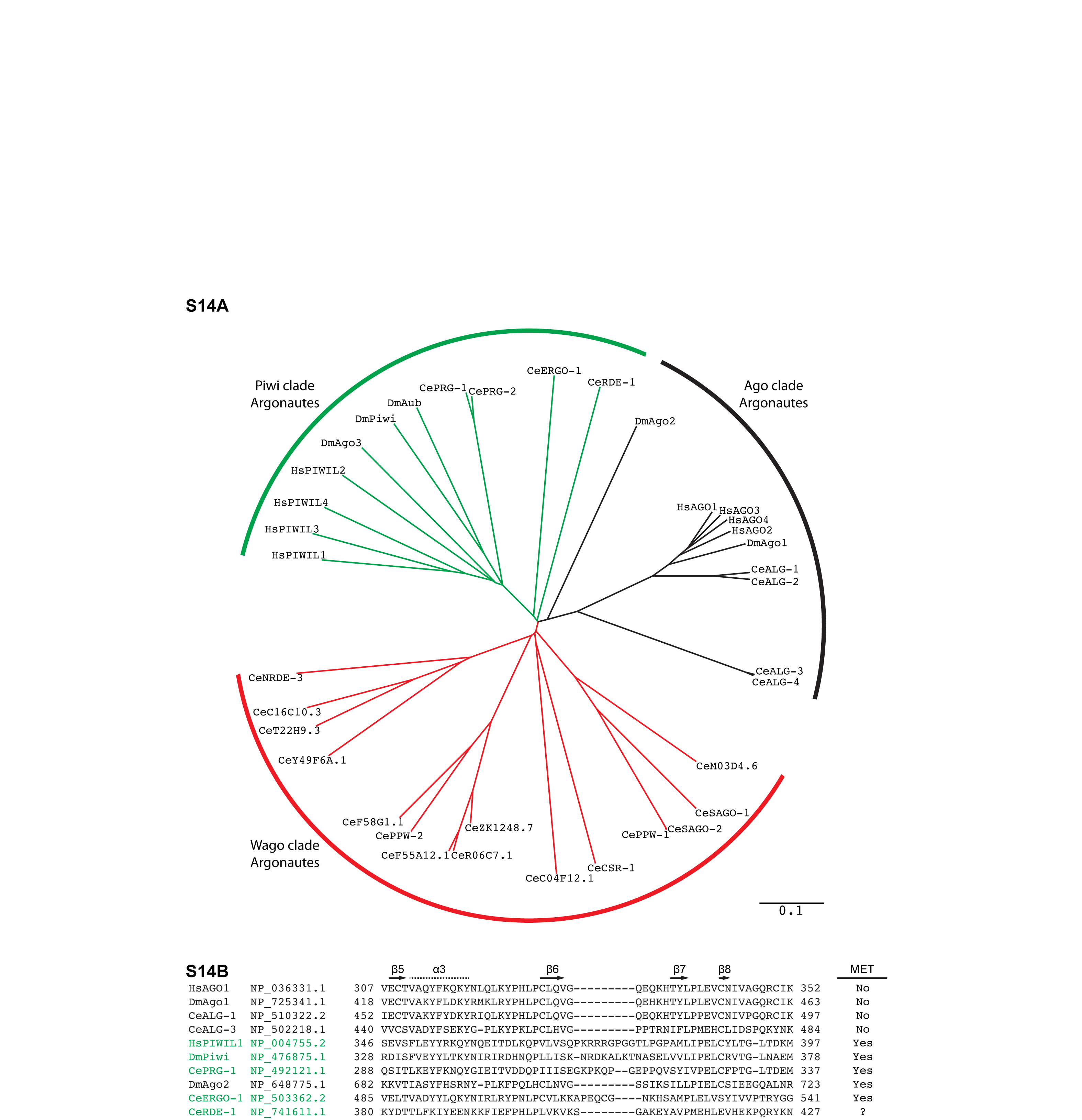

Supplement: Figure S14 — Comparison of C. elegans Argonautes. A) Phylogram of human, fly, and worm Argonautes shows divergence of CeERGO-1, CeRDE-1, and DmAgo2 relative to other members of their clades. Multiple sequence alignment of the longest annotated RefSeq protein sequences was performed using ClustalW with default parameters and visualized using Phylodendron (version 0.8d; http://www.es.embnet.org/Doc/phylodendron/). Scale, 0.1∶ 0.1 substitutions per site. B) Only Piwi clade Argonautes bear the characteristic PAZ domain insertion. Multiple sequence alignment of select Argonautes was performed using ClustalW with default parameters and cropped to show the context of the PAZ domain insertion between strands β6 and β7 as annotated by Tian et al. [69]. For each Argonaute, methylation status (MET) of associated small RNAs is indicated at right (Yes, methylated; No, not methylated). Sources: HsAGO1, [68], [69] and by analogy to mouse [31]; DmAgo1, [19]; CeALG-1, [19]; CeALG-3, this study; HsPIWIL1, [69] and by analogy to mouse [30], [31]; DmPiwi, [31], [32]; CePRG-1, [27] and this study, DmAgo2, [9], [22]; CeERGO-1, [27], [42] and this study. (TIF) [file pgen.1002617.s014.tif]
