# Supplementary material for: The Caenorhabditis elegans HEN1 Ortholog, HENN-1, Methylates and Stabilizes Select Subclasses of Germline Small RNAs
Source: PLoS Genet. 2012 Apr 19;8(4):e1002617. doi: 10.1371/journal.pgen.1002617 (PMC3330095; doi:10.1371/journal.pgen.1002617)
Supplement: Table S1 — Oligonucleotides for Northern Blot Analysis. Oligonucleotides corresponding to the antisense sequences of small RNAs were synthesized by Integrated DNA Technologies and used for small RNA detection by northern blot. (DOC) [file pgen.1002617.s015.doc]

**Table S1: Oligonucleotides for northern blot analysis**

| **Small RNA target** | **Probe sequence** |
| --- | --- |
| cel-miR-1 | 5’ TACATACTTCTTTACATTCCA /3StarFire/ 3’ |
| 21UR-845 | 5’ ttcgagttcttgctttcctga /3StarFire/ 3’ |
| 21UR-4292 | 5’ ccattctttgtcaccctcgta /3StarFire/ 3’ |
| 21UR-4748 | 5’ tagccagtactctacgttgta /3StarFire/ 3’ |
| 21UR-5941 | 5’ attaaccgttcgtgccccgaa /3StarFire/ 3’ |
| 26G-O1 | 5' TTGAAAATAATCTACCGTTTCTGAGC /3StarFire/ 3' |
| 26G-O3 | 5' AAAAGTATCCGACTTTCGAGTTTGTC /3StarFire/ 3' |
| 26G-O7 | 5’ ttccacgatcagaagggatgtcactc /3StarFire/ 3’ |
| 26G-O8 | 5’ tgctgcgaaaactgtggatttcctac /3StarFire/ 3’ |
| 26G-S5 | 5' TACCATGTCGCTCACTGCTGATCCAC /3StarFire/ 3' |
| 26G-S7 | 5' cgatgatcatattctacttcattttc /3StarFire/ 3' |
