# Supplementary material for: The Caenorhabditis elegans HEN1 Ortholog, HENN-1, Methylates and Stabilizes Select Subclasses of Germline Small RNAs
Source: PLoS Genet. 2012 Apr 19;8(4):e1002617. doi: 10.1371/journal.pgen.1002617 (PMC3330095; doi:10.1371/journal.pgen.1002617)
Supplement: Table S2 — Small RNA Sequences for Taqman Probe Design. Sequences of the indicated small RNAs were submitted to Applied Biosystems for Taqman small RNA probe design and synthesis. (DOC) [file pgen.1002617.s016.doc]

**Table S2: Small RNA sequences for Taqman probe design**

| **Small RNA** | **Sequence (5’ to 3’)** |
| --- | --- |
| let-7 | tgaggtagtaggttgtatagtt |
| miR-1 | TGGAATGTAAAGAAGTATGTA |
| miR-124 | taaggcacgcggtgaatgcca |
| 21UR-845 | TCAGGAAAGCAAGAACTCGAA |
| 21UR-1063 | TGAGCGCATTTGTATACACTG |
| 21UR-1267 | Taggaacgaaatgaacaaaat |
| 21UR-1343 | TGAAGGAAGAGTACGAAACTT |
| 21UR-1832 | TTTAACAAATGACGGTAAATC |
| 21UR-1838 | Ttgttcttcgttcggtccaaa |
| 21UR-1848 | TAAAGGCAGAATTTTATCAAC |
| 21UR-3129 | TGTATGTAAAACTTTACGGCA |
| 21UR-5191 | TGTAAAAAGTTTTTTGATGTA |
| 22G targeting *Tc3* | GAATCAGAACCAGTCTGGAGAT |
| 22G targeting *E01G4.7* | gagtgacatcccttctgatcgt |
| 26G-O1 | GCTCAGAAACGGTAGATTATTTTCAA |
| 26G-O3 | GACAAACTCGAAAGTCGGATACTTTT |
| 26G-O4 | GAGGGGATAAGAGCTCGTCCGATGGC |
| 26G-O5 | GATGGGAATGCAGAAGAAAAGAGGGG |
| 26G-O6 | GTAGAAGGATTCATCTGGCATTTCAT |
| 26G-O7 | GAGTGACATCCCTTCTGATCGTGGAA |
| 26G-O8 | GATGAATCGTCGATAGAAAGACAAAC |
| 26G-O9 | GTAGGAAATCCACAGTTTTCGCAGCA |
| 26G-S1 | GCTATGGAGGACGAGAATACATAATT |
| 26G-S5 | GTGGATCAGCAGTGAGCGACATGGTA |
| 26G-S6 | GACTCTTCGACTTCGGCATTTGCGGA |
| 26G-S7 | GAAGAACGAAAATTTGAAGATGTATA |
| 26G-S8 | GAAAATGAAGTAGAATATGATCATCG |
