# Supplementary material for: The Caenorhabditis elegans HEN1 Ortholog, HENN-1, Methylates and Stabilizes Select Subclasses of Germline Small RNAs
Source: PLoS Genet. 2012 Apr 19;8(4):e1002617. doi: 10.1371/journal.pgen.1002617 (PMC3330095; doi:10.1371/journal.pgen.1002617)
Supplement: Table S3 — Primers for RT-qPCR. RT-qPCR primers for detection of the indicated gene targets were synthesized by Integrated DNA Technologies. (DOC) [file pgen.1002617.s017.doc]

**Table S3:** Primers for RT-qPCR

| **Gene target** | **Forward (5’ to 3’)** | **Reverse (5’ to 3’)** |
| --- | --- | --- |
| *act-1* | CCAGGAATTGCTGATCGTATGCAGAA | TGGAGAGGGAAGCGAGGATAGA |
| *C40A11.10* | AATGGCTCCTTGAAAAGATCG | TACATTTCCGCCACGTTGAAA |
| *E01G4.7* | GCACAAGGTTTCGTTCTTGGTG | AGTGACATCCCTTCTGATCG |
| *eft-2* | TGTGTTTCCGGAGTGTGTGT | CCATCGTCGTCTCCGTAAGT |
| *F39E9.7* | CCCAGTGGCCCAATTAAACG | GCACAAGGTTTCGTTCTTGGTG |
| *F55C9.5* | ACCATTGGAGCACGTAAATCAA | GGTCCTAATAATAAAGTTGCGTCG |
| *fbxa-65* | ACTTACAAGGATCAAGAAAAGCG | CCTTGACCGCTATTCCGAGAAA |
| *fbxb-37* | CATAAGTCCTGGAAGCCATACTCC | ATCTTTCGATACGATGTATGTTCG |
| *K02E2.6* | CAGTGGTACAAGTGGGAGTAAACG | AATTGGCAAGTAACTGATTCCG |
| *henn-1* | GGAAGAGGGATGTGTTCAACG | TTCCAGTGCTGATGCGATCATA |
| *ssp-16* | GTCATCAAACAACAATGAGTACCG | GCTCCAGCAGTGCGAGTGAT |
| *T05E12.8* | TTCCATTTGAGGATTTTGCTACG | ATTATTTGGATGGCAGCCGATG |
| *Tc3* | GAGCGTTCACGGAGAAGAAG | AATAGTCGCGGGTTGAGTTG |
| *Y82E9BR.20* | CTCCCGCTTTCTTGATGTATTG | AGTCCGAACTCATCCAAAGCAG |
